# Eco-Focused Menu Labels on Full Meal Orders From Fast-Food Restaurants: A Randomized Clinical Trial

**DOI:** 10.1001/jamahealthforum.2026.2108

**Published:** 2026-07-10

**Authors:** Julia A. Wolfson, Anna Claire Tucker, Alexandria E. Reimold, Aviva Musicus, Nina Carr, Brent F. Kim, Raychel Santo, Clara Cho, Daphne Abigail Barquera Guevara, Erin Choi, Cindy W. Leung, Christina A. Roberto, Jennifer Falbe

**Affiliations:** 1 Department of International Health, Johns Hopkins Bloomberg School of Public Health, Baltimore, Maryland; 2 Department of Health Policy and Management, Johns Hopkins Bloomberg School of Public Health, Baltimore, Maryland; 3 Department of Human Ecology, University of California, Davis; 4 Department of Nutrition, Harvard T.H. Chan School of Public Health, Boston, Massachusetts; 5 Johns Hopkins Center for a Livable Future, Johns Hopkins Bloomberg School of Public Health, Baltimore, Maryland; 6 Department of Environmental Health & Engineering, Johns Hopkins Bloomberg School of Public Health, Baltimore, Maryland; 7 World Resources Institute, Washington, DC; 8 Perelman School of Medicine, University of Pennsylvania, Philadelphia; 9 Center for Science in the Public Interest, Washington, DC

## Abstract

**Importance:**

Promoting environmentally sustainable dietary patterns can benefit both population and planetary health. Ecolabels on restaurant menus could promote healthful and sustainable choices, but evidence is needed on effects in different restaurant types.

**Objective:**

To test the effects of different ecolabel designs on menus from 2 fast-food restaurants on the healthfulness of full meal orders, compared to a control label.

**Design, Setting, and Participants:**

This randomized clinical trial was conducted online in a nationally representative sample of adults (≥18 years) in the US in April 2025. Participants were recruited from the NORC AmeriSpeak survey panel to participate in this experiment.

**Intervention:**

Participants were randomized via an automated process within the survey platform to 1 of 5 label conditions: (1) control: QR code; (2) low–climate-impact labels; (3) traffic-light labels (TLLs); (4) high–climate-impact labels; or (5) grade-scale labels and asked to order lunch from 2 fast-food menus (1 burger-focused and 1 sandwich-focused).

**Main Outcomes and Measures:**

The primary outcome was the healthfulness of meals ordered based on a modified Nutrient Profile Index (NPI) score. Other outcomes included the estimated greenhouse gas emissions (GHGE), calorie and nutrient content, and cost of selected meals, as well as perceptions of labels and knowledge about the climate contribution of different foods.

**Results:**

Among the 6210 participants (3274 [51.0%] women; mean [SD] age, 48.1 [18.1] years), 1236 participants were randomized to the control group; 1249 to low–climate-impact labels, 1243 to high–climate-impact labels, 1235 to TLLs; and 1247 to grade-scale labels. Demographic characteristics were similar across groups. Only the TLL was effective at promoting more healthful food choices in the burger restaurant compared to the control label (mean [standard error] NPI score: 49.93 [0.18] points vs 49.25 [0.18] points; *P* < .001). There were no ecolabel effects on NPI score in the sandwich restaurant. Compared to control, the TLL led to 15.2% lower GHGE for meals in the burger restaurant and 29.8% lower GHGE for meals in the sandwich restaurant. The TLL was the most noticeable (78.30% [95% CI, 75.35%-81.26%]; *P* < .001) and had the highest percentage of participants who correctly reported what the label was about (88.72% [95% CI, 86.04%-91.39%]; *P* < .001). There were no differences in the overall cost of the meals selected by label condition.

**Conclusions and Relevance:**

Findings from this web-based randomized clinical trial showed that TLL-style ecolabels on restaurant menus may be an effective strategy to promote more sustainable and, in some instances, more healthful food choices without increasing consumer costs.

**Trial Registration:**

ClinicalTrials.gov Identifier: NCT06909019

## Key Points

**Question:**

What effects do ecolabel designs have on the healthfulness and environmental sustainability of full meal orders at 2 fast-food restaurants in a nationally representative sample?

**Findings:**

In this randomized clinical trial with 6210 adults in the US, the traffic-light label outperformed other label designs and the control label. The traffic-light label led to meal selections with 30% lower greenhouse gas emissions from a sandwich restaurant and 15% lower greenhouse gas emissions and slightly more healthful meals ordered from a burger restaurant compared to the control label.

**Meaning:**

Traffic-light–style ecolabels may be an effective strategy to promote more sustainable choices from fast-food restaurants.

## Teaser

This randomized clinical trial tests the effects of different ecolabel designs on menus from 2 fast-food restaurants on the healthfulness of full meal orders, compared to a control label.

## Introduction

The food system,^1,2^ especially red meat production,^2,3^ is a large contributor to global greenhouse gas emissions (GHGE) and climate change. Red and processed meat consumption is also associated with numerous adverse health outcomes, with stronger evidence for processed meat.^4,5,6,7,8^ In contrast, more sustainable diets are associated with better diet quality.^2,9,10,11,12^ Therefore, promoting more environmentally sustainable dietary patterns is a key strategy that can benefit both population and planetary health.

On a typical day, one-third of adults in the US eat fast food, a major source of red meat in the US diet.^13,14,15^ Therefore, even small shifts toward more sustainable menu offerings and consumer choices in fast-food restaurants could lead to meaningful improvements in diet quality and human and planetary health. Sustainability is a trend in the restaurant sector,^16,17,18^ and numerous food brands have begun voluntarily implementing ecolabeling schemes.^18,19,20^ It is important to understand how different ecolabel designs affect both the healthfulness and sustainability of consumer food choices, particularly in fast-food restaurants, where most menu items are not healthful.^21,22,23^

Ecolabels may promote more healthful and/or more sustainable food selections.^24,25,26,27,28,29,30^ However, in restaurant settings, the few ecolabel studies to date have not assessed full meal orders,^26^ have not compared label effects across different restaurant types,^25,26,30^ and only one (using a nonrepresentative sample) has directly compared different label designs^30^ (eg, warning labels, grade-scale labels [eg, A-F] or traffic-light labels [TLL; ie, red, yellow, or green]).^31,32,33^ Ecolabels may increase perceptions of the healthfulness of items labeled as sustainable,^26,34,35^ whether that perception is justified or not. Further, perceptions of labeled items and efficacy of labels may differ depending on menu composition and restaurant type.

This study’s objective was to test the effect of different ecolabels on the healthfulness of full meal orders in 2 fast-food restaurants, modeled after popular burger- and sandwich-focused chains. Secondary objectives were to assess label effects on GHGE, calories, and nutrients of selected meals, and understanding and perceptions of the ecolabels and labeled menu items. Given existing evidence,^31,32,33^ we hypothesized that, relative to a control label, the TLL would lead to the most healthful food choices, followed by the high-impact label, then the grade-scale label.

## Methods

### Study Design and Participants

This randomized clinical trial followed the Consolidated Standards of Reporting Trials (CONSORT) reporting guideline. This trial was approved by the Johns Hopkins Bloomberg School of Public Health Institutional Review Board. Written informed consent was obtained from all participants. The study was conducted from April 1 to April 23, 2025. Analyses were preregistered on AsPredicted.com (219376).

Participants were recruited from the National Opinion Research Center (NORC) AmeriSpeak panel, a probability-based, nationally representative panel of US households recruited by telephone, mail, and field interviews.^36^ Adults in the US (aged ≥18 years) were eligible for this study. The target sample size was 6000 based on a priori power calculations to ensure a small detectable effect (Cohen *d* = 0.14) with greater than 80% power for the primary outcome. The trial protocol can be found in Supplement 1.

### Procedures

Participants were invited to complete a survey for a research study “to understand things you consider when ordering food from a fast-food restaurant.” First, participants were instructed to order lunch from each restaurant. Participants were randomized 1:1:1:1:1 to view menus with one of the following label conditions: (1) control: QR code labels on all items; (2) low–climate-impact labels on select items; (3) TLLs (red, yellow, or green) on all items; (4) high–climate-impact labels on select items; or (5) grade-scale labels (A-F) on all items (eAppendix 4 in Supplement 2). Two menus based on popular burger- and sandwich-based fast-food restaurant chains were presented to participants in random order and were labeled according to participants’ assigned label condition. Menus also displayed prices (extracted and averaged from the restaurants’ websites in 16 US cities (eAppendix 1 in Supplement 2), calorie information (per US regulations), and explanatory text at the top describing the meaning of the labels. Participants were required to choose 1 main item and could separately select up to 3 additional items from a section displaying sides, beverages, and desserts. To encourage realistic choices, we used a minor deception: participants were informed that 1 in 25 respondents would receive a coupon for the meals they select, in addition to their survey-completion compensation. Participants were compensated in AmeriSpeak points, which can be redeemed for prizes. Those who “won” the raffle were compensated with additional points equal to the value of their selected meals. eTable 1 in Supplement 2 lists all items on the 2 menus. After ordering, participants completed a survey.

### Measures

#### Healthfulness of the Selected Meal

The primary outcome was the mean Nutrition Profile Index (NPI) score of the selected meal. NPI was selected because it has been widely used to measure healthfulness of fast-food restaurant menu items,^37,38,39,40,41^ and can be calculated using publicly available nutrition information provided by restaurants. NPI is based on the United Kingdom Ofcom Nutrient Profiling Model, which assigns individual foods a score from 0 to 100, with 64 or higher considered healthful.^39^ Points are awarded for positive components (ie, fiber, protein, fruit, vegetables, and nuts) and subtracted for negative components (ie, calories, saturated fat, sodium, and sugar). Further details on calculating NPI score are available elsewhere.^39,42^ We obtained nutritional and serving size information from the restaurant websites (eAppendix 1 in Supplement 2). Because NPI was originally developed to score individual foods, we used a modified NPI score to score full meals, calculating the mean NPI score of all foods in the meal weighted by the mass of each food. Since NPI scoring does not differentiate beverages well, beverages were excluded from mean NPI meal scores and assessed separately as a secondary outcome. Secondary outcomes were preregistered and are described below. eTable 2 in Supplement 2 describes item wording and response options.

#### GHGE of the Selected Meal

GHGE, kilograms carbon dioxide equivalent (kgCO_2_e) per meal, were calculated by multiplying the estimated quantity of each ingredient in a menu item by an associated GHGE factor from the Coolfood Pledge and Coolfood Meals Calculators,^43,44^ then summing the result over all ingredients in all menu items in each meal. GHGE factors included supply chain emissions and carbon opportunity costs. Beverages were excluded from meal-level GHGE due to limitations in accurately assessing their GHGE. Following methods described by Waite and Blondin,^45^ thresholds for labels (eg, yellow vs green TLLs) were set relative to average meal-related GHGE, and the GHGE reductions needed to align with climate mitigation goals. Further description of GHGE methods and associated thresholds are available in eAppendix 2 in Supplement 2. We also assessed whether participants selected a sustainable (ie, assigned green TLL) main entrée item (eg, burger, sandwich).

#### Perceptions of Labeled Items

We showed participants 4 menu items (labeled according to condition) from each restaurant representing 4 permutations of high/low healthfulness and high/low sustainability and asked about perceptions of appeal, healthfulness, and the climate impact of each item. The order of the restaurant and items within each restaurant were randomized. Responses were measured on 7-point Likert scales.

#### Perceived Message Effectiveness

Participants viewed the label corresponding to their assigned condition and answered questions about perceived message effectiveness using a modified version of the University of North Carolina Perceived Message Effectiveness (PME) scale.^46,47^ The modified PME included 4 items, and scores ranged from 1 to 5 for each item, with higher scores indicating greater message effectiveness. Scores were averaged across the 4 items to create a mean PME scale. We further assessed label believability and the extent to which it grabs attention. All responses were measured on a 5-item unipolar scale.

#### Noticeability and Label Use

Participants were asked if they “notice any label (other than calories).” Those who reported noticing the ecolabel were asked what the label was about and whether they used the label when deciding what to order.

#### Knowledge of Climate Impact

Participants were asked to rank the climate impact of 3 foods (tofu, beef, and chicken) from smallest to largest climate impact. We also asked participants to determine which of 2 items from each restaurant (beef burger vs chicken sandwich; ham sandwich vs roast beef sandwich) had the larger climate impact or whether it was the same. In each question, participants were shown images of the menu items in random order and labeled according to condition. The outcome was the percentage of participants responding correctly to both questions.

#### Total Nutrients of Selected Meal

We assessed the total energy (calories) and nutrient content (saturated fat [g], protein [g], fiber [g], total sugar [g], and sodium [mg]) of selected meals (including beverages). Additional outcomes included NPI score of the main item selected, red meat selection (yes/no), sugar-sweetened beverage selection (yes/no), and total cost of selected meal, including beverages.

#### Sociodemographic Characteristics

Self-reported sociodemographic characteristics were part of participants’ profiles and were provided by AmeriSpeak. Race categories included Hispanic, non-Hispanic Black, non-Hispanic White, non-Hispanic Other, non-Hispanic multiracial, and non-Hispanic Asian/Pacific Islander. Due to small sample sizes in some categories, a category for other was created to include non-Hispanic with other race, multiracial, and Asian/Pacific Islander.

### Statistical Analysis

We used AmeriSpeak’s study-specific survey weights to obtain nationally representative estimates. For primary analyses, we used linear regression models with label condition as the independent variable to estimate the predicted mean NPI score with standard error (SE), stratified by restaurant. We included an interaction term between label condition and whether the burger menu or sandwich menu was presented first in models with a significant interaction. To examine the predicted mean total kgCO_2_e per meal stratified by restaurant, we used generalized linear models with a gamma family and log link, as GHGE were positively skewed. We tested primary models for interaction by sociodemographic characteristics (sex, age, race and ethnicity, education, income, region, metropolitan/nonmetropolitan residence, and political beliefs), and noticing a label. Linear models with experimental condition as the independent variable were also used to estimate PME, attitudes, beliefs, mean cost, energy, and nutrients of selected meals, stratified by restaurant. We used logistic regression, stratified by restaurant, to estimate the following probabilities by experimental condition: noticing a label, correctly identifying the label, using the label, correctly identifying menu items with higher climate impact, correctly ranking foods from lowest to highest climate impact, selecting a sustainable item, selecting an item containing red meat, and selecting a sugar-sweetened beverage. We used postestimation margins commands to obtain predicted means and probabilities. All analyses were conducted in Stata 18 statistical software (StataCorp LLC). We considered statistical significance at *P* < .05 with Bonferroni-Holm correction for pairwise comparisons of the 4 label conditions for main analyses including NPI score and GHGE, and within family of outcomes for secondary outcomes. All tests were 2-sided.

## Results

**Figure 1.**
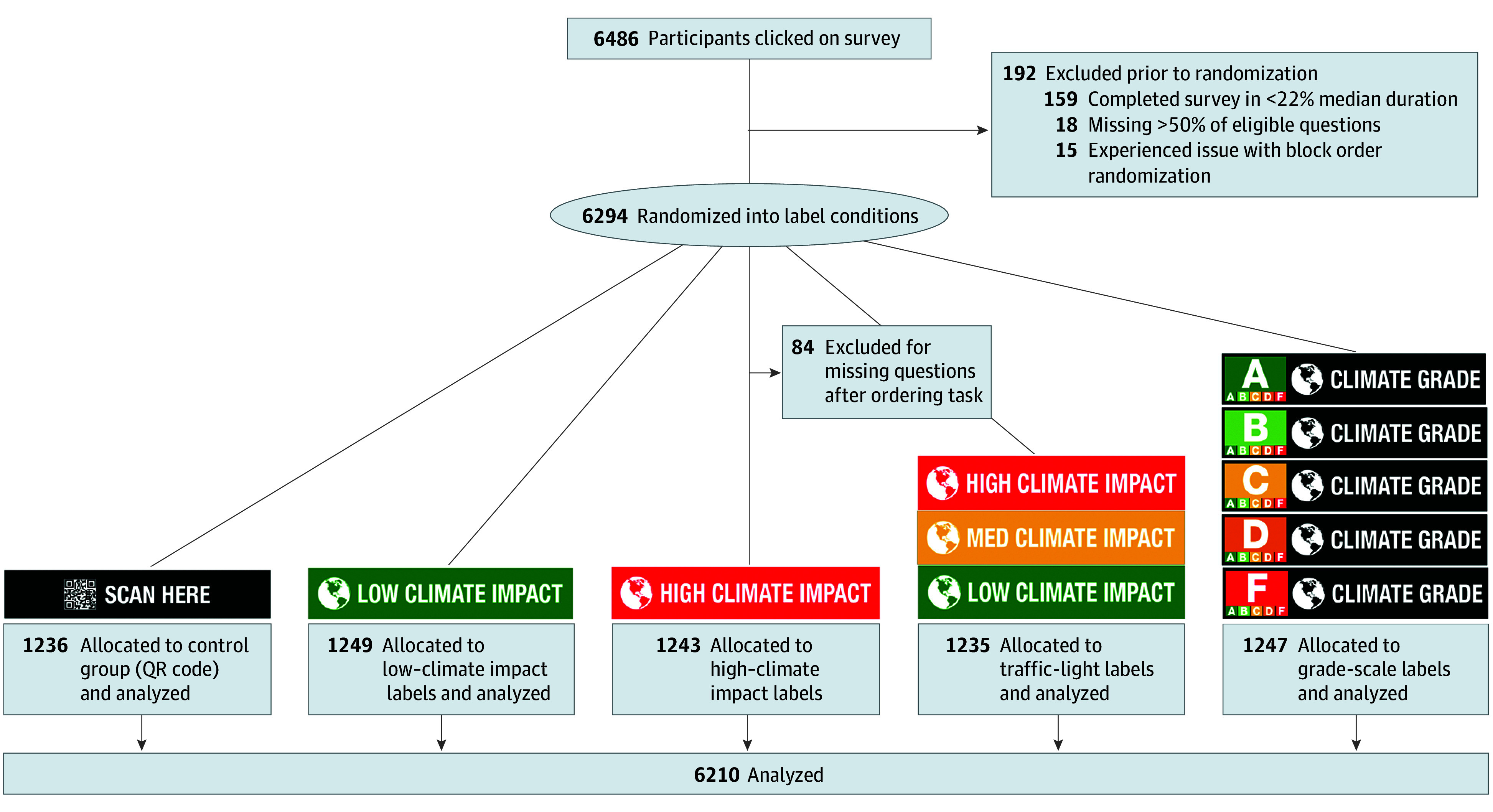
CONSORT Flow Diagram

Of the 6486 participants who started the survey, 15 participants were excluded due to issues with block order randomization (not preregistered). Additional preregistered exclusions included completing the survey in less than 33% of the median survey duration (n = 159), missing greater than 50% of eligible questions (n = 18), and missing responses after the menu ordering tasks (n = 84) (Figure 1).

**Figure 2.**
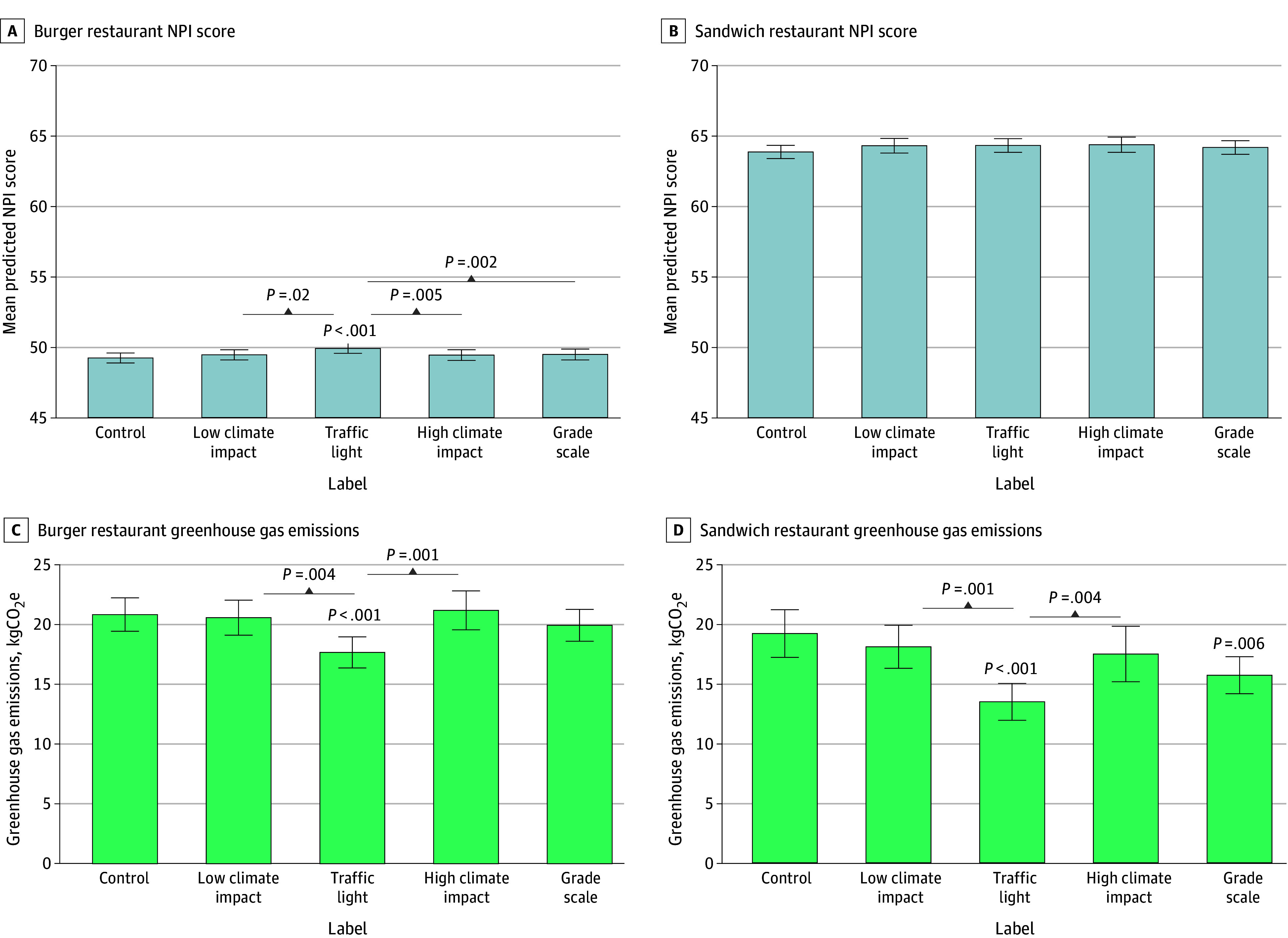
Bar Chart of Mean Predicted Nutrition Profile Index (NPI) Score and Greenhouse Gas Emissions of Meals by Experimental Condition and Restaurant (N = 6210) Statistical significance was set at *P* < .05 with Bonferroni-Holm correction for multiple comparisons of 4 label conditions. Meals exclude beverages. A, Postestimation margins after weighted simple linear regression and including an interaction term for the order restaurant menus were presented. B, Postestimation margins after weighted simple linear regression. C and D, Postestimation margins after weighted generalized linear model with a gamma family and log link. The error bars indicate 95% CIs.

The final analytic sample included 6210 participants (3274 [51.0%; 95% CI, 49.4%-52.7%] women and 2936 [49.0%; 95% CI, 47.3%-50.6%] men; mean [SD] age 48.1 [18.1] years; 783 [12.0%; 95% CI, 11.0%-13.1%] Black, 1128 [17.8%; 95% CI, 16.5%-19.2%] Hispanic, 3854 [61.0%; 95% CI, 59.3%-62.6%] White, and 445 [9.2%; 95% CI, 8.2%-10.3%] had another race) (Table 1). Frequency of menu item selections overall and across label conditions is presented in eTable 3 in Supplement 2. In the burger-based restaurant, across experimental arms there were differences in frequency of selecting the plant-based burger with the lowest frequency in the control condition (3.4%; 95% CI, 2.4%-4.7%; *P* < .001) and the highest frequency in the TLL condition (9.8%; 95% CI, 7.7%-12.5%; *P* < .001). In the burger-based restaurant, individuals in the TLL condition selected more healthful meals than those in the control (mean [SE] NPI score, 49.93 [0.18] vs 49.25 [0.18], respectively; *P* < .001), and the high-impact (mean [SE] NPI score, 49.45 [0.20]; *P* = .005) and grade-scale (mean [SE] NPI score, 49.50 [0.20]; *P* = .002) conditions (Figure 2). There were no significant differences in selections of individual menu items (eTable 3 in Supplement 2) or in NPI scores (Figure 2) by label condition in the sandwich-based restaurant. There were no significant differences in mean calories or nutrients by label condition in either restaurant (eTable 4 in Supplement 2). NPI scores for the entrée selected are reported in eFigure 2 in Supplement 2. There were no significant interactions between label conditions and sociodemographic characteristics in NPI scores in either restaurant.

**Table 1.** Participant Characteristics Overall and by Experimental Condition

Characteristic	Label, No. (weighted %) [95% CI]					
	Overall	Control	Low climate impact	Traffic light	High climate impact	Grade scale
Participants	6210 (100)	1236 (19.9)	1249 (20.1)	1235 (19.9)	1243 (20.0)	1247 (20.1)
Sex						
Female	3274 (51.0) [49.4-52.7]	658 (52.2) [48.6-55.9]	663 (50.8) [47.1-54.5]	671 (53.1) [49.4-56.7]	629 (48.6) [44.9-52.3]	653 (50.5) [46.7-54.2]
Male	2936 (49.0) [47.3-50.6]	578 (47.8) [44.1-51.4]	586 (49.2) [45.5-52.9]	564 (46.9) [43.3-50.6]	614 (51.4) [47.7-55.1]	594 (49.5) [45.8-53.3]
Age group, y						
18-29	961 (19.6) [18.2-21.1]	191 (19.1) [16.2-22.4]	181 (18.6) [15.6-22.1]	182 (19.2) [16.1-22.7]	214 (21.3) [18.2-24.7]	193 (19.8) [16.6-23.4]
30-44	1751 (25.7) [24.4-27.1]	337 (25.5) [22.5-28.9]	349 (24.1) [21.3-27.2]	368 (27.5) [24.5-30.7]	338 (24.4) [21.4-27.6]	359 (27.1) [24.1-30.4]
45-59	1437 (23.8) [22.4-25.2]	280 (23.1) [20.3-26.2]	295 (24.9) [21.8-28.1]	295 (23.4) [20.5-26.5]	272 (23.8) [20.7-27.3]	295 (23.7) [20.7-27.0]
≥60	2061 (30.9) [29.4-32.4]	428 (32.2) [28.9-35.7]	424 (32.4) [29.1-35.8]	390 (29.9) [26.7-33.3]	419 (30.5) [27.3-33.9]	400 (29.4) [26.2-32.7]
Race and ethnicity						
Hispanic	1128 (17.8) [16.5-19.2]	232 (19.6) [16.8-22.8]	216 (17.5) [14.8-20.6]	216 (18.3) [15.5-21.5]	222 (16.0) [13.5-19.0]	242 (17.6) [14.8-20.8]
Non-Hispanic Black	783 (12.0) [11.0-13.1]	159 (12.8) [10.6-15.4]	170 (13.0) [10.7-15.6]	147 (11.9) [9.7-14.6]	144 (10.3) [8.4-12.6]	163 (12.1) [9.9-14.5]
Non-Hispanic White	3854 (61.0) [59.3-62.6]	770 (60.8) [57.1-64.3]	769 (59.4) [55.7-63.0]	784 (61.6) [57.9-65.1]	791 (64.0) [60.4-67.6]	740 (59.1) [55.3-62.8]
Other^a^	445 (9.2) [8.2-10.3]	75 (6.8) [5.0-9.0]	94 (10.1) [7.9-12.9]	88 (8.1) [6.2-10.6]	86 (9.6) [7.3-12.5]	102 (11.2) [8.7-14.3]
Education						
<High school	358 (8.9) [7.9-10.2]	76 (9.9) [7.6-12.8]	71 (8.3) [6.3-10.9]	77 (9.8) [7.5-12.7]	70 (8.6) [6.4-11.4]	64 (8.3) [5.9-11.5]
High school graduate or equivalent	1139 (28.7) [27.1-30.3]	240 (30.1) [26.6-33.8]	225 (28.6) [25.1-32.5]	218 (27.6) [24.2-31.3]	237 (30.4) [26.8-34.2]	219 (26.7) [23.2-30.4]
Some college or associate’s degree	2418 (26.5) [25.2-27.8]	473 (25.8) [23.1-28.7]	500 (27.8) [25.0-30.9]	482 (26.1) [23.4-28.9]	480 (26.4) [23.6-29.3]	483 (26.3) [23.5-29.2]
Bachelor’s degree	1341 (22.5) [21.2-23.9]	246 (20.5) [17.8-23.6]	264 (21.3) [18.5-24.3]	278 (23.7) [20.8-26.8]	275 (23.3) [20.3-26.6]	278 (23.8) [20.9-27.0]
Postgraduate study or professional degree	954 (13.4) [12.4-14.4]	201 (13.7) [11.7-16.1]	189 (13.9) [11.7-16.4]	180 (12.9) [10.8-15.3]	181 (11.4) [9.5-13.5]	203 (15.0) [12.7-17.6]
Household income, $						
<30 000	1306 (21.0) [19.7-22.4]	274 (22.0) [19.1-25.3]	261 (19.6) [16.9-22.7]	264 (21.4) [18.5-24.6]	260 (22.1) [19.1-25.5]	247 (20.0) [17.1-23.2]
30 000 to <60 000	1508 (23.9) [22.5-25.3]	307 (25.9) [22.9-29.2]	320 (25.6) [22.5-28.8]	299 (23.7) [20.7-26.9]	298 (23.3) [20.4-26.6]	284 (20.8) [18.1-23.9]
60 000 to <100 000	1548 (24.6) [23.2-26.0]	319 (25.0) [22.0-28.3]	312 (24.8) [21.7-28.0]	307 (24.9) [21.9-28.2]	292 (22.6) [19.7-25.8]	318 (25.7) [22.6-29.1]
≥100 000	1848 (30.5) [29.0-32.1]	336 (27.0) [23.9-30.3]	356 (30.1) [26.8-33.6]	365 (30.0) [26.8-33.4]	393 (31.9) [28.5-35.5]	398 (33.5) [30.0-37.2]
Region						
Northeast	827 (17.3) [16.0-18.6]	155 (16.3) [13.7-19.3]	172 (18.4) [15.6-21.6]	160 (16.6) [13.9-19.7]	171 (17.6) [14.8-20.7]	169 (17.5) [14.7-20.7]
Midwest	1691 (20.5) [19.3-21.7]	347 (21.7) [19.1-24.6]	361 (21.4) [18.7-24.3]	341 (20.6) [18.0-23.4]	316 (18.7) [16.3-21.4]	326 (20.0) [17.5-22.9]
South	2099 (38.4) [36.8-40.1]	438 (40.9) [37.4-44.6]	397 (37.0) [33.5-40.6]	417 (38.9) [35.4-42.6]	443 (40.3) [36.6-44.0]	404 (35.2) [31.7-38.8]
West	1593 (23.6) [22.4-25.3]	296 (21.1) [18.2-24.2]	319 (23.3) [20.3-26.5]	317 (23.9) [21.0-27.0]	313 (23.4) [20.4-26.7]	348 (27.3) [24.0-30.9]
Metropolitan residence						
Nonmetropolitan	879 (13.5) [12.4-14.6]	195 (15.5) [13.1-18.2]	179 (13.1) [10.9-15.7]	183 (14.0) [11.8-16.6]	159 (12.6) [10.5-15.1]	163 (12.2) [10.1-14.7]
Metropolitan	5331 (86.5) [85.4-87.6]	1041 (84.5) [81.8-86.9]	1070 (86.9) [84.3-89.1]	1052 (86.0) [83.4-88.2]	1084 (87.4) [84.9-89.5]	1084 (87.8) [85.3-89.9]
Political beliefs						
Unknown	43 (0.7) [0.5-1.1]	5 (0.5) [0.2-1.3]	6 (0.5) [0.2-1.7]	12 (1.1) [0.5-2.5]	9 (0.7) [0.3-1.6]	11 (0.9) [0.4-1.7]
Very liberal	817 (12.1) [11.1-13.2]	181 (14.1) [11.7-16.9]	162 (11.8) [9.8-14.3]	163 (12.4) [10.3-14.8]	156 (10.1) [8.3-12.3]	155 (12.1) [9.9-14.7]
Somewhat liberal	662 (10.4) [9.4-11.5]	137 (10.5) [8.5-12.8]	137 (10.8) [8.7-13.4]	133 (10.2) [8.1-12.7]	128 (9.6) [7.7-11.9]	127 (10.7) [8.3-13.8]
Moderate	3052 (49.8) [48.1-51.4]	588 (49.1) [45.4-52.7]	605 (49.3) [45.7-53.0]	610 (49.1) [45.5-52.8]	623 (52.8) [49.1-56.5]	626 (48.4) [44.7-52.2]
Somewhat conservative	872 (14.7) [13.6-15.9]	175 (14.4) [12.0-17.1]	159 (13.2) [10.9-16.0]	173 (15.0) [12.6-17.8]	170 (13.9) [11.6-16.6]	195 (17.2) [14.5-20.2]
Very conservative	764 (12.3) [11.3-13.4]	150 (11.5) [9.5-13.8]	180 (14.2) [11.9-17.0]	144 (12.2) [10.0-14.8]	157 (12.8) [10.5-15.5]	133 (10.7) [8.6-13.2]

^a^ Due to small sample sizes in some categories, the other group was created to include non-Hispanic with other race, multiracial, and Asian/Pacific Islander.

In both restaurants, participants in the TLL condition selected meals with lower GHGE than the control condition (burger restaurant: 15.2% lower; mean [SE], 17.9 [0.7] vs 21.1 [0.7] kgCO2e; *P* = .001; sandwich restaurant: 29.8% lower; mean [SE], 13.6 [0.8] vs 19.4 [1.0] kgCO2e; *P* < .001) (Figure 2). In both restaurants, the TLL condition also had lower GHGE compared to the low-impact condition (burger restaurant: mean [SE], 17.9 [0.7] vs 20.1 [0.7] kgCO2e; sandwich restaurant: mean [SE], 13.6 [0.8] vs 18.3 [0.9] kgCO2e, respectively) and the high-impact condition (burger restaurant: mean [SE], 17.9 [0.7] vs. 21.4 [0.8] kgCO2e; sandwich restaurant: mean [SE], 13.6 [0.8] vs. 17.6 [1.2] kgCO2e, respectively). Compared to the control, the TLL condition also had higher probability of selecting a sustainable (green-labeled) item in both restaurants (eFigure 3 in Supplement 2). In the burger restaurant, the TLL condition had a lower probability of selecting a red meat item compared to the control, low-impact, and high-impact conditions (eFigure 4 in Supplement 2).

The TLL was the most noticeable (78.30% [95% CI, 75.35%-81.26%]; *P* < .001) compared to the control (42.85% [95% CI, 39.23%-46.48%]) (eTable 5 in Supplement 2) and had the highest percentage of participants who correctly reported what the label was about (88.72% [95% CI, 86.04%-91.39%]; *P* < .001). Less than half of participants reported using the ecolabels when ordering; the highest percentage was in the TLL condition (38.9% [95% CI, 34.5%-43.2%]). Differences by label condition in the healthfulness of the meal orders did not differ based on label noticeability (eFigure 1 in Supplement 2).

Table 2 presents perceptions of 4 menu items in each restaurant by label condition. In both restaurants, the TLLs and the grade-scale labels were significantly different than the control label in rankings of the climate impact of all menu items, with lower and higher GHGE items correctly perceived as having lower and higher climate impact, respectively.

**Table 2.** Mean Predicted Participant Perceptions of Menu Items at a Burger-Focused and Sandwich-Focused Restaurant by Treatment Condition (N = 6210)^a^

Menu item	Control, mean (95% CI) (n = 1236)	Label							
		Low climate impact (n = 1249)		Traffic light (n = 1235)		High climate impact (n = 1243)		Grade scale (n = 1247)	
		Mean (95% CI)	*P* value	Mean (95% CI)	*P* value	Mean (95% CI)	*P* value	Mean (95% CI)	*P* value
**How appealing do you think this menu item is?** ^b^									
Burger restaurant									
Plant-based burger	4.02 (3.88-4.16)	3.76 (3.62-3.91)	.01	3.86 (3.71-4.01)	.12	3.80 (3.65-3.94)	.03	3.80 (3.65-3.94)	.03
8-Piece chicken nuggets	4.40 (4.28-4.52)	4.18 (4.05-4.31)	.02	4.35 (4.22-4.48)	.63	4.28 (4.15-4.40)	.17	4.32 (4.20-4.44)	.37
Burger	5.00 (4.88-5.12)	4.79 (4.67-4.91)	.02	4.84 (4.72-4.96)	.07	4.82 (4.70-4.95)	.04	4.82 (4.70-4.94)	.04
Bacon burger	4.65 (4.51-4.79)	4.40 (4.26-4.54)	.01	4.49 (4.35-4.63)	.12	4.45 (4.31-4.60)	.06	4.38 (4.23-4.52)	.009
Sandwich restaurant									
6-in Tuna	3.89 (3.75-4.04)	3.97 (3.83-4.10)	.46	4.08 (3.94-4.22)	.06	3.88 (3.74-4.02)	.93	3.88 (3.74-4.02)	.91
Grilled chicken salad	5.09 (4.99-5.20)	4.95 (4.84-5.06)	.07	4.99 (4.87-5.11)	.20	4.96 (4.84-5.07)	.09	4.99 (4.88-5.10)	.18
Roast beef salad	4.10 (3.97-4.23)	4.10 (3.97-4.23)	.99	3.92 (3.79-4.06)	.06	3.95 (3.82-4.07)	.09	3.92 (3.79-4.05)	.06
12-in Steak and cheese	4.90 (4.78-5.02)	4.83 (4.70-4.97)	.46	4.67 (4.54-4.80)	.01	4.69 (4.55-4.83)	.02	4.71 (4.58-4.85)	.04
**How healthy do you think this item is?** ^c^									
Burger restaurant									
Plant-based burger	3.53 (3.42-3.65)	3.73 (3.61-3.84)	.02	3.85 (3.73-3.97)	<.001	3.65 (3.54-3.76)	0.15	3.78 (3.65-3.90)	0.005
8-Piece chicken nuggets	2.87 (2.78-2.97)	2.97 (2.87-3.07)	.17	3.07 (2.98-3.17)	.004	2.87 (2.77-2.96)	0.92	3.07 (2.97-3.17)	0.005
Burger	2.80 (2.71-2.90)	2.74 (2.65-2.83)	.35	2.56 (2.47-2.65)	<.001	2.75 (2.66-2.85)	0.48	2.70 (2.60-2.80)	0.15
Bacon burger	2.21 (2.11-2.30)	2.12 (2.03-2.22)	.21	2.02 (1.93-2.11)	.005	2.16 (2.07-2.26)	0.52	2.06 (1.96-2.16)	0.03
Sandwich restaurant									
6-in Tuna	4.46 (4.35-4.56)	4.53 (4.43-4.64)	.31	4.63 (4.53-4.73)	.02	4.48 (4.38-4.57)	0.79	4.54 (4.45-4.64)	0.24
Grilled chicken salad	5.31 (5.22-5.39)	5.30 (5.21-5.39)	.89	5.34 (5.25-5.44)	.55	5.27 (5.19-5.36)	0.61	5.26 (5.17-5.35)	0.48
Roast beef salad	4.52 (4.42-4.62)	4.54 (4.43-4.64)	.83	4.12 (4.02-4.23)	<.001	4.25 (4.15-4.35)	<0.001	4.33 (4.22-4.44)	0.01
12-in Steak and cheese	3.21 (3.11-3.31)	3.15 (3.05-3.24)	.39	2.93 (2.83-3.03)	<.001	3.07 (2.97-3.16)	0.05	3.00 (2.89-3.10)	0.004
**What do you think the climate impact of this item is?** ^d^									
Burger restaurant									
Plant-based burger	4.41 (4.31-4.51)	4.60 (4.52-4.69)	.003	4.74 (4.66-4.83)	<.001	4.48 (4.38-4.57)	.35	5.01 (4.89-5.13)	<.001
8-Piece chicken nuggets	4.27 (4.17-4.37)	4.44 (4.35-4.53)	.02	4.56 (4.48-4.64)	<.001	4.38 (4.29-4.46)	.13	4.53 (4.44-4.62)	<.001
Burger	4.12 (4.02-4.22)	3.95 (3.85-4.04)	.02	3.20 (3.11-3.29)	<.001	3.41 (3.31-3.51)	<.001	3.46 (3.36-3.57)	<.001
Bacon burger	3.89 (3.78-4.00)	3.60 (3.50-3.71)	<.001	2.97 (2.88-3.07)	<.001	3.16 (3.06-3.26)	<.001	2.76 (2.62-2.89)	<.001
Sandwich restaurant									
6-in Tuna	4.49 (4.40-4.59)	4.62 (4.54-4.70)	.04	4.80 (4.72-4.88)	<.001	4.63 (4.55-4.72)	.03	4.73 (4.65-4.82)	<.001
Grilled chicken salad	4.63 (4.54-4.72)	4.81 (4.73-4.89)	.004	4.93 (4.87-5.00)	<.001	4.81 (4.73-4.89)	.005	4.88 (4.79-4.96)	<.001
Roast beef salad	4.39 (4.29-4.48)	4.28 (4.19-4.37)	.11	3.45 (3.36-3.54)	<.001	3.58 (3.49-3.67)	<.001	3.74 (3.63-3.84)	<.001
12-in Steak and cheese	4.17 (4.06-4.27)	3.93 (3.84-4.02)	.001	3.22 (3.13-3.31)	<.001	3.39 (3.29-3.48)	<.001	2.98 (2.85-3.12)	<.001

^a^ Mean predicted scores from postestimation margins obtained from weighted simple linear regression.

^b^ Rated on a 7-point scale: 1 = very unappealing; 2 = unappealing; 3 = slightly unappealing; 4 = neither appealing nor unappealing; 5 = slightly appealing; 6 = appealing; 7 = very appealing.

^c^ Rated on a 7-point scale: 1 = very unhealthy; 2 = unhealthy; 3 = slightly unhealthy; 4 = neither healthy nor unhealthy; 5 = slightly healthy; 6 = healthy; 7 = very healthy.

^d^ Rated on a 7-point scale: 1 = extremely high climate impact; 2 = very high; 3 = high; 4 = moderate; 5 = low; 6 = very low; 7 = extremely low.

Compared to the control label, all ecolabels were less believable but had higher PME, and only the high-impact and the TLL were ranked higher for grabbing attention (Table 3). The TLL was ranked highest for believability (3.11 [95% CI, 3.03-3.19]) other than the control label (3.56 [95% CI, 3.48-3.63]) and had the highest PME of all the labels (2.62 [95% CI, 2.54-2.71]).

**Table 3.** Mean Predicted Attitudes and Beliefs About Labels by Treatment Condition (N = 6210)^a^^,^^b^

Attitude/belief	Control, mean (95% CI) (n = 1236)	Low climate impact (n = 1249)		Traffic light (n = 1235)		High climate impact (n = 1243)		Grade scale (n = 1247)	
		Mean (95% CI)	*P* value	Mean (95% CI)	*P* value	Mean (95% CI)	*P* value	Mean (95% CI)	*P* value
This label is believable to me.	3.56 (3.48-3.63)	2.80 (2.71-2.88)	<.001	3.11 (3.03-3.19)	<.001	2.99 (2.90-3.08)	<.001	3.05 (2.96-3.14)	<.001
This label grabs my attention.	2.96 (2.88-3.04)	2.74 (2.66-2.83)	<.001	3.20 (3.12-3.29)	<.001	3.21 (3.12-3.30)	<.001	2.99 (2.90-3.08)	.678
Mean perceived message effectiveness^c^	1.88 (1.80-1.95)	2.22 (2.15-2.29)	<.001	2.62 (2.54-2.71)	<.001	2.60 (2.51-2.69)	<.001	2.56 (2.48-2.65)	<.001
This label discourages me from wanting to eat foods with a high climate impact.	1.82 (1.74-1.91)	2.11 (2.03-2.20)	<.001	2.53 (2.43-2.62)	<.001	2.54 (2.44-2.63)	<.001	2.48 (2.39-2.58)	<.001
This label makes eating foods with a high climate impact seem unpleasant.	1.84 (1.76-1.92)	2.15 (2.06-2.23)	<.001	2.61 (2.51-2.70)	<.001	2.63 (2.53-2.73)	<.001	2.57 (2.47-2.67)	<.001
This label makes me concerned about the health effects of eating menu items with a high climate impact.	1.95 (1.87-2.04)	2.25 (2.16-2.34)	<.001	2.62 (2.52-2.71)	<.001	2.55 (2.45-2.65)	<.001	2.56 (2.46-2.65)	<.001
This label makes me concerned about the environmental effects of eating menu items with a high climate impact.	1.89 (1.80-1.98)	2.37 (2.28-2.46)	<.001	2.74 (2.65-2.83)	<.001	2.68 (2.58-2.78)	<.001	2.65 (2.55-2.75)	<.001

^a^ Mean predicted scores from postestimation margins obtained from weighted simple linear regression.

^b^ Rated on a 5-point scale: 1 = not at all; 2 = a little bit; 3 = somewhat; 4 = quite a bit; 5 = a great deal.

^c^ Mean score from the 4 Perceived Message Effectiveness questions.

Participants in all ecolabel conditions were more likely to correctly identify menu items with higher climate impact compared to the control with the highest proportion of correct responses in the TLL condition (66.07% correct [95% CI, 62.64%-69.50%]) (eTable 6 in Supplement 2). There were no differences in probability of selecting a sugar-sweetened beverage (eFigure 5 in Supplement 2), and no differences in the overall cost of the meals selected (eFigure 6 in Supplement 2) by label condition in either restaurant.

## Discussion

In this web-based randomized clinical trial with a large, nationally representative sample, we found that TLL-style ecolabels in which items receive a red, yellow, or green label led to more healthful meal selections in a burger-focused fast-food restaurant and lower climate impact (GHGE) meal selections in both burger- and sandwich-focused restaurants. While the magnitude of differences in the healthfulness of meal selections compared to the control and other ecolabels was small, and less than 50% of participants said they used the labels, the TLL consistently and meaningfully performed better than the control and other ecolabels across multiple outcomes related to sustainability, label noticeability and understanding, knowledge about healthfulness and climate impact. Meal cost did not differ across label conditions. Results signal that TLL-style menu ecolabels may be effective at promoting more sustainable food choices in fast-food restaurants while also educating consumers about how food choices contribute to climate change. TLLs may also promote more healthful food choices in some restaurants without incurring extra costs to consumers or adversely impacting fast-food restaurants’ profits.

These results align with prior research showing the effectiveness of interpretive food/nutrition labels,^30,32^ TLL (that provide information about high- and low-impact items simultaneously) being most effective, and showing small effects of ecolabels on nutrition outcomes.^25,26,30^ Compared to the control condition, the TLL led to 15.2% and 29.8% lower GHGE for meals ordered from the burger restaurant and sandwich restaurant, respectively. Given the scale and popularity of large chain restaurants,^13,14^ reductions of GHGE of this magnitude could make a meaningful cumulative difference in addressing climate change and should be considered as part of multifaceted strategies to promote more sustainable food industry practices and consumer choices.

While consumers have indicated that sustainable food choices are important to them, public awareness of the role of reducing red meat intake to mitigate climate change is low.^48,49^ The 2025 to 2030 Dietary Guidelines for Americans’ increased emphasis on promoting red meat may shape public perceptions and complicate efforts to align dietary behaviors with sustainability goals.^50^ The present results signal that ecolabels on restaurant menus may increase knowledge and awareness about the climate impact of menu items, particularly red meat items compared to other protein sources, even though fewer than half used the labels in their food selections, despite noticing and accurately reporting what the labels conveyed. Ultimately, ecolabels may impact consumer behavior and food choices at the time of purchase but also in the future and, perhaps, in other venues beyond fast-food restaurants^19,24,25,33^; however, more research is needed in retail and restaurant settings and with repeated exposures to labels to explore these possibilities further.

Whether ecolabel effects would vary depending on restaurant type has been a key question. We found that, in both restaurants, the TLL led to more sustainable food selections. However, we found differential effects on the healthfulness of selections. As in prior research, differences in meal healthfulness scores and nutrition outcomes were small in both restaurants, likely due, in part, to the limited range of nutrition values across menu items.^25,26,30^ It will be important for future research to investigate whether ecolabels could have a stronger effect on meal selection healthfulness when tested in restaurants with greater variation in menu items’ nutritional profiles.

Food and menu labels are a feasible policy that can prompt supply-side changes via reformulation or menu composition and, in some instances, can shift consumer knowledge and behavior.^31,33,51,52,53,54,55,56^ Ecolabels may use these same mechanisms to reduce food systems-related climate change. Internationally, ecolabels have garnered interest from governments.^57,58^ In the US, though there had previously been some federal interest in pursuing climate food labels,^59^ there is currently more promise for state- or local-level policy. Further, in the absence of local, state, or federal policy, restaurants and food companies can take voluntary action to implement ecolabels, which some have already done.^60,61,62^ Mandatory labels, with a consistent, evidence-based design, would be helpful to limit consumer confusion and prompt greater industry and consumer behavior change.

### Limitations

The primary limitation of this study is that it was a hypothetical, 1-time ordering task simulation in which participants did not order food for actual consumption and did not spend their own money, which limits generalizability. Although the study’s purpose was masked, social desirability bias may still be a concern. As with other nutrition scoring systems, the NPI measure has limitations. NPI scores are applied at the item, rather than ingredient or meal level. Additionally, NPI scoring does not differentiate beverages well. The modified meal-level NPI (weighted mean of food item scores), attempts to address these limitations. The limited range of menu items and nutrition values across menu items, and the per–100-g basis of the primary outcome (NPI score), which does not differentiate portion sizes, may have limited our ability to see differences in these outcomes. Additionally, while our calculations of climate impact focus on GHGE, impacts on biodiversity, water use, soil health, and pollution are also important sustainability considerations. Future research is needed to test ecolabels in restaurants with greater heterogeneity in nutrition and climate impact of menu items.

## Conclusions

This randomized clinical trial demonstrated that traffic-light–style menu ecolabels may be an effective strategy to promote more sustainable food choices without increasing consumer costs. In some restaurants, ecolabels may also lead to more healthful food choices; however, more research is needed, particularly in different types of restaurants.

## Notes

Supplement 1. Trial Protocol

Supplement 2. eAppendix 1. Nutrition and Price Information Methods Appendix eAppendix 2. Greenhouse Gas Emissions Methods Appendix eAppendix 3. Results Appendix. eTable 1. Nutrition Profile Index, Calories, and Greenhouse Gas Emissions for Menu Items Shown to Participants eTable 2. Secondary Outcomes wording and response options eTable 3. Menu Item Selection Frequency (n [%]) by Experimental Condition (N=6210) eTable 4. Mean Predicted Nutrients of Full Meal Ordered by Experimental Condition and Restaurant (N=6210) eFigure 1. Mean Predicted Nutrition Profile Index Score of Meal Selected at the Burger Restaurant (Panel A) and the Sandwich Restaurant (Panel B) by Experimental Condition and Noticeability (n=6209) eTable 5. Mean Predicted Probabilities of Noticing Label, Identifying What Label was About, and Label Use by Treatment Condition eTable 6. Mean Predicted Probabilities of Correctly Identifying Higher Climate Impact Menu Items and Ranking Items by Climate Impact by Treatment Condition (n=6242) eFigure 2. Mean Predicted Nutrition Profile Index Score of Selected Main Item at the Burger Restaurant (Panel A) and the Sandwich Restaurant (Panel B) by Treatment Condition (n=6210) eFigure 3. Predicted Probability of Selecting Sustainable Item at the Burger Restaurant (Panel A) and the Sandwich Restaurant (Panel B) by Treatment Condition (n=6210) eFigure 4. Predicted Probability of Selecting Item Containing Red Meat the Burger Restaurant (Panel A) and the Sandwich Restaurant (Panel B) by Treatment Condition (n=6210) eFigure 5. Predicted Probability of Selecting a Sugar Sweetened Beverage at the Burger Restaurant (Panel A) and the Sandwich Restaurant (Panel B) by Treatment Condition (n=6210) eFigure 6. Mean Predicted Cost of selected Meals at the Burger Restaurant (Panel A) and the Sandwich Restaurant (Panel B) by Treatment Condition (n=6210) eTable 7. Summary Table of Key Results Across Label Conditions eAppendix 4. Menu images for different label conditions

Supplement 3. Data Sharing Statement
