# Errors in Figure 1

**DOI:** 10.1001/jamahealthforum.2026.3064

**Published:** 2026-08-07

In the Original Investigation titled “Eco-Focused Menu Labels on Full Meal Orders From Fast-Food Restaurants: A Randomized Clinical Trial,”^1^ published online on July 10, 2026, the CONSORT diagram in Figure 1 did not contain corresponding label images included with menu items. The images were instead described in the Figure caption. This article has been corrected online.
