# Supplementary material for: Eco-Focused Menu Labels on Full Meal Orders From Fast-Food Restaurants: A Randomized Clinical Trial
Source: JAMA Health Forum. 2026 Jul 10;7(7):e262108. doi: 10.1001/jamahealthforum.2026.2108 (PMC13355149; doi:10.1001/jamahealthforum.2026.2108)
Supplement: Supplement 1. — Trial Protocol [file jamahealthforum-e262108-s001.pdf]

BSPH IRB Research Plan for New Data Collection

IRB Version: 01Sep2023

For new data collection, new data collection plus secondary data analysis, biospecimen repositories, and data coordinating center protocols.

DO NOT DELETE ANY QUESTIONS FROM THIS TEMPLATE

PI Name: Julia Wolfson

Study Title: The Menu Labels for Environmental Impact and Nutrition Study (MENU Study): Online Experiment

IRB No.: IRB00027451

PI Version No. / Date: Verson 3/ 01172024

I. **Aims of the Study**: *Describe the aims/objectives of the research and/or the project's research questions or hypotheses.*

Aim 1: Compare the effects of different climate-impact menu label designs on the healthfulness of fast-food meal orders in a nationally representative sample of US adults. In an online experiment, we will randomly assign 6,000 adults to view menus from two fast-food restaurants (one burger, one sandwich) that display one of five types of climate-impact label designs: 1) control (QR code); 2) green 'low climate impact' label; 3) red 'high climate impact' label; 4) 'climate-score' label; and 5) traffic-light label. Our large and national sample will enable us to explore differences in effects by self-reported gender, age, education, income, region, and political views. We *hypothesize* that 'high climate impact' labels will be most effective, followed by traffic-light labels, at improving the healthfulness (Nutrient Profile Index (NPI) score) of restaurant meal selections.

II. **Background and Rationale**: *Explain why this study is being done. Summarize briefly what is already known about the issue and reference previously published research, if relevant.*

Shifting dietary patterns in the United States (US) toward more environmentally sustainable 'planetary health' diets has the potential to improve dietary quality and prevent nutrition-related chronic diseases like obesity and type-2 diabetes. Fast-food restaurants are a key environment to promote 'planetary health'-aligned food choices given the frequency of fast-food consumption, the poor dietary quality of fast-foods, and their associations with adverse health outcomes. Numerous restaurants and food companies have recently implemented climate-impact labels that inform consumers of each menu item's carbon footprint (i.e., climate impact). Climate-impact menu labels are also garnering interest among policymakers. Several European countries are implementing a national climate label for food; there is federal- and state-level policy interest in the US; and multiple universities, hospitals, and workplaces have voluntarily adopted climate-labeling systems in food service settings. However, evidence is lacking on how climate-impact labels affect the healthfulness of dietary intake. Existing research shows that *nutrition* labels can change behavior, but we lack evidence on whether *climate* labels can promote healthier as well as more climate friendly food choices. Climate labels could improve dietary quality via lower red and processed meat intake, but could also promote undeserved perceptions that unhealthy food items are

healthy (i.e., a ‘health-halo’ effect). Given the rapid development and growing food industry and policy interest in climate-impact labels, there is a *critical need* for timely, rigorous evaluation of the real-world effects of climate labels in different types of restaurants, and the optimal design to maximize behavior change. There is a particular need to examine the longer-term effects of climate-impact labels on dietary quality beyond a one-time exposure.

III. Study Design:

A. *Provide a BRIEF overview of your study design and methods. The study design must relate to your stated aims/objectives. DETAILS WILL BE REQUESTED LATER. If your study also involves analysis of existing data, please complete Section XI, “Secondary Data Analysis of Existing Data” in the last part of this research plan. If your study ONLY involves analysis of existing data, please use the research plan template for secondary data analysis (JHSPH IRB Research Plan for Secondary Data Analysis of Existing Data/Specimens).*

The *objective* of this aim is to evaluate the degree to which four different types of climate-impact labels influence the healthfulness of fast-food orders compared to a control label (QR code). To accomplish this aim, we will recruit a large, nationally representative sample of U.S. adults (N=6,000) to participate in a randomized, controlled experiment. Participants will be randomized to view two restaurant menus (one popular burger chain and one popular sandwich chain) that display either the control label or one of the four climate-impact labels (a green low-climate-impact label, a red ‘high-climate-impact’ warning label, a ‘climate-score’ label, or a traffic light label). Participants will then order a meal for dinner from each restaurant that they have a chance to actually receive if they are selected via a lottery. After ordering, participants will complete a 10-minute survey to assess label perceptions and sociodemographic characteristics.

B. *Provide a sample size and a justification as to how you arrived at that number. If you use screening procedures to arrive at a final sample, distinguish the screening sample size from the enrolled sample size; a table may be helpful. For electronic survey studies involving online recruitment and survey completion: consider how you will set controls on how many people will join your study.*

For primary analyses, our sample size of 6,000, 1,200 per experimental condition, provides 80% power to detect Cohen’s d=0.14, a small effect size, with a conservative Bonferroni adjusted alpha of 0.0125 to account for multiple comparisons (4 experimental conditions each compared to control). This effect size is small compared to the magnitude of the effects we saw in our preliminary study of climate-impact labels in an online fast-food restaurant. In that study the limited menu had little variation in the NPI scores of items, however we still saw an effect size of Cohen’s d=0.21 for the difference in NPI scores of items ordered between the ‘high climate impact’ label and the control condition. The broader literature on randomized trials of other menu labels on continuous outcomes,[34,35] including a meta-analysis of SSB warning labels[95] finds larger effects than we are powered to detect in this study (Cohen’s d=0.32 for hypothetical behavioral outcomes and Cohen’s d=0.17 for actual behavioral outcomes). For environmental outcomes, based on our preliminary study in which there was a 23% difference between the ‘high climate impact’ label and control in the proportion of participants ordering a sustainable item, the minimum detectable effect between label and control conditions is 6.8% at alpha=0.0125 and 80% power.[1] Additionally, a recent randomized online experiment of a GHGE label, found an effect size of Cohen’s d=0.50.[39] In that study, participants in the GHGE label condition made more sustainable food choices compared to the control group. Therefore, we will have adequate power to detect small effects between labels.

C. *Does your study meet the NIH definition of “clinical trial”:* ““**A research study in which one or more human subjects are prospectively assigned to one or more interventions** (which may include placebo or other control) to evaluate the effects of those interventions on health-related biomedical or behavioral outcomes”?

If yes, the study must be listed on clinicaltrials.gov, study personnel must complete GCP training, and federally funded studies must post consent forms on approved sites, like clinicaltrials.gov.

Yes. The study will be listed on clinicaltrials.gov prior to beginning any data collection.

IV. Participants:

Describe the study participants and the population from which they will be drawn. Specify the inclusion and exclusion criteria. If you plan to include children, note their ages and whether you will include children in foster care or who are wards of the State. Note if the participants are particularly vulnerable in terms of cognitive limitations, education, legal migration status, incarceration, poverty, or some combination of factors.

A. Inclusion Criteria:

1. Member of the NORC AmeriSpeak panel
2. aged ≥18 years and

B. Exclusion Criteria:

1. Not a member of the NORC AmeriSpeak panel
2. <18 years old

***NOTE**: If you are recruiting participants or receiving, accessing, or using data from a U.S. health care provider, HIPAA review is likely to be required. If you plan to bring identifiable health information from a foreign country to a U.S. covered entity (e.g., lab at the Hopkins SOM), HIPAA may be triggered. Check "yes" to the HIPAA question in the PHIRST application.*

V. Study Procedures:

In this section, provide details of your procedures, particularly as they relate to human subjects. If this is a multi-center study, make the role of JHSPH clear. If you will collaborate with other institutions or organizations, or plan to subcontract JHSPH responsibilities to others, make clear their responsibilities in the Study Oversight section of this document. Be aware that all recipients of federal funding for non-exempt human subjects research must have a Federal Wide Assurance (FWA) , which is a promise to comply with human subjects research regulations.

*If the JHSPH will serve as **data coordinating center**, indicate in the sections below which procedures JHSPH will not be performing. Additional information regarding data coordinating centers is requested in a later section.*

If your study will develop in phases, address each item below by phase.

A. Recruitment Process:

1. *Describe how you will identify, approach, and inform potential participants about your study. Include details about who will perform these activities and their qualifications.*

   We will recruit a nationally representative sample of 6,000 US adults through the NORC AmeriSpeak panel.[69] The NORC AmeriSpeak panel is a nationally representative probability-based panel used frequently in academic research. Households are selected randomly, with a known, non-zero probability of selection, from a sampling frame that represents >97% of the US population. The current panel size includes >54,000 individuals in >43,000 households. The AmeriSpeak panel includes an oversampling of Hispanic and African American household and households with lower socioeconomic status and employs robust methods to recruit and retain panelists from underrepresented groups. Panelists typically complete 1-2 surveys per month. Our team has extensive experience fielding and analyzing large experimental surveys such as the one proposed here. We have previously worked with NORC AmeriSpeak on a study of climate-impact menu

labels, and data were collected in about two weeks. Following data collection, NORC AmeriSpeak provided clean datasets with survey weights to produce nationally representative estimates within three weeks. Therefore, we anticipate data collection for this aim to be complete in one month.

2. *Address any* <u>*privacy*</u> *issues associated with recruitment. If recruitment itself may put potential participants at risk (if study topic is sensitive, or study population may be stigmatized), explain how you will minimize these risks*.

   There are no privacy issues associated with recruitment. The survey will be distributed to NORC AmeriSpeak panel members by NORC staff. The research team will have no direct interaction with participants and will only receive deidentified data.

B. **<u>Consent Process</u>**:

1. *Describe the following details about obtaining informed consent from study participants. If a screening process precedes study enrollment, also describe the consent for screening*.

   a. *Who will obtain informed consent, and their qualifications*:

NORC AmeriSpeak panel participants provide consent when they enroll in the panel. For surveys that do not cover sensitive topics study-specific consent is not generally required.

   b. *How, where, and when the consent discussion(s) will occur*:

      NORC AmeriSpeak panel participants provide consent when they enroll in the panel.

   c. *The process for determining whether a potential participant meets eligibility criteria. If you will collect personally identifiable information for screening purposes, collect only data needed for this purpose and explain what will happen to the data for individuals who are not eligible*:

      NORC staff will determine if study participants are eligible to participate based on demographic information in the panel member's profile.

   d. *Whether you will obtain a signature from the participant or will use an oral consent process*:

      No.

   e. *Whether you will obtain a legally authorized representative's signature for adults lacking capacity*:

      N/A

   f. *If children are included in the study, if and how you will obtain assent from them*:

      N/A

   g. *If children are included in the study, how you will obtain permission for them to participate from their parent, legal guardian, or other legal authority (if child is in foster care or under government supervision). If any of the children are "wards of the state", additional regulatory requirements will apply:*

      N/A

   h. *If you are seeking a waiver of informed consent or assent, the justification for this request*:

      N/A

   i. *Whether you will include a witness to the consent process and why*:

      We will not include a witness to consent process.

j. *If the language is unwritten, explain how you will communicate accurate information to potential participants and whether you will use props or audio materials*:

N/A

2. Identify the countries where the research will take place, and the languages that will be used for the consent process.

Country	Consent Document(s) (Adult Consent, Parental Permission, Youth Assent, etc.)	Languages
USA	Adult consent has already been granted by AmeriSpeak panel members.	English

C. **Study Implementation**:

1. *Describe the procedures that participants will undergo. If complex, insert a table below to help the reviewer navigate*.

Randomization. Participants will be randomized to one of five labeling conditions: 1) control (QR code) label, shown on all food items; 2) green 'low climate-impact' label, shown on all low climate-impact food items; 3) red 'high climate-impact' warning label, shown on all high-climate-impact food items; 4) 'climate-score' label, shown on all food items (one of five 'grades' will be shown for each labeled item'; and 5) traffic light label, with one of three levels shown on all food items. All label conditions will also display 1) a statement at the top of the menus describing the meaning of label in the condition and 2) calorie labels, as is required by law in all large chain restaurants in the US. We selected our climate-impact labels based on our preliminary research, which demonstrated that the low and high climate-impact labels encouraged people to reduce their likelihood of selecting a beef menu item.[1] Our third climate label is based on the Nutri-Score labeling system in France that displays a letter grade to reflect the nutritional quality of food. Emerging evidence supports this labeling system, which is appealing for its intuitive design.[48,70,71] Our fourth label is based on studies showing that traffic light nutrition labels increase purchases of 'green' foods and reduce purchases of 'red' foods.[72,73]

Restaurant ordering procedure: We will program two realistic fast-food restaurant menus, modeled after Burger King and Jimmy John's, into Qualtrics, as we have done in prior studies. Participants will be instructed to imagine they are ordering a meal for themselves for dinner. They will view and order from the two realistic fast-food menus presented in random order that will display the labeling scheme to which the participant has been randomized. Participants will select the items they want to order for their meal from each of the restaurant menus and then complete a brief survey that will include questions about the meals, the labels, and their attitudes about climate change and healthy eating. To encourage realistic food selections and enhance external validity, we will use a minor deception procedure that has been approved by the IRB for other labeling studies we have completed. At the beginning of the study, participants will be told that 1 in 25 individuals will be selected via lottery to receive a coupon for the actual items in the meal they ordered. In reality, upon completing the study, participants who won the lottery (for each meal) will be provided with compensation equivalent to the amount they spent on their meal rather than being restricted to a coupon for the meal. After being debriefed about the study purpose, all participants will have the ability to indicate whether they want their

data to be used. In our studies that use this procedure, it is extremely rare for someone to not allow us to use their data after the study debriefing.

Survey procedure: After the restaurant menu ordering task, participants will complete a brief, 10-minute survey designed to assess the degree to which the labels influence the potential mechanisms of behavior change that we identified in our conceptual framework. First, to determine whether the climate-impact labels may lead consumers to infer that low climate-impact items are healthier (even if that is not the case), we will show participants four images of low climate-impact menu items that do not meet the NPI threshold for "healthy" (two from the burger restaurant menu and two from the sandwich restaurant menu). Participants will rate these items on 5-point Likert scales designed to assess how **healthy** and **tasty** participants perceive the items to be. Second, participants will then be asked if they **noticed** the climate-impact labels on the restaurant menus. Third, we will ask participants questions to assess their **knowledge** of the climate-impact of their food choices. Fourth, participants will then answer two questions about their environmental **values** and their perceived **subjective norms** about climate change. Finally, participants will be shown the label to which they were randomized and we will assess their 1) **emotional reaction** to it, and 2) the **perceived message effectiveness,** which has been shown to predict consumer behavior.[81,82] See **Table 1** for example survey items.

2. *Describe the number and type of study visits and/or contacts between the study team and the participant, how long they will last, and where/how they will take place*.

   Participants will participate in one online survey in which they will select items to hypothetically order from two fast food restaurants. Total participation will be <10 minutes. The participant can access the survey from their computer at a time and place of their choosing.

3. *Describe the expected duration of the study from the perspective of the individual participant and duration overall*.

   <10 minutes.

4. *Provide a brief data analysis plan and a description of variables to be derived.*

Outcomes: The primary dietary outcome will be the healthfulness of the fast-food meal selections, measured with the Nutrient Profile Index (NPI) score. NPI scores are based on the UK Ofcom Nutrient Profiling Model, which is used to score foods in the U.K. to determine which ones can be marketed to children. The NPI [84]generates a 0-100 point score for foods and beverages where scores ≥64 are considered healthy.[89-91] NPI scores are based on points awarded from nutrients to encourage (e.g., percent fruits and vegetables, grams of fiber and protein) and nutrients of concern (e.g., sodium, sugar) per 100 grams. The NPI has been validated[92] and used previously to quantify the nutrition quality of chain restaurant menu items and packaged food items in the US and United Kingdom.[55,89,93] We chose NPI score rather than other dietary quality indices because it is the most feasible given publicly available nutrition and serving size information and we have used it in prior research in large chain restaurants.[55] Our other secondary dietary outcomes will include the total energy (kcal), grams of saturated fat, sugar, protein, fiber, and milligrams of sodium ordered at each restaurant and whether the meal contains a red meat item (yes/no). In addition, we will examine the climate impact of the menu choices by calculating the GHGE of the meal selections using the Cool Food Calculator method described above. Our other knowledge and perception secondary outcomes are described above.

Statistical analyses: To determine the effects of the four climate-impact labels compared to the control label on our continuous primary outcome of dietary quality, we will regress the outcome onto a categorical indicator representing experimental condition. If the primary outcome is not normally distributed we will perform appropriate transformations and use robust standard error estimates to preserve valid standard errors and p-values. We will use this approach for secondary kcal and GHGE outcomes. For dichotomous secondary outcomes, we will use Poisson regression with a robust error variance to directly estimate the probability ratio (i.e., risk ratio).[94] This method is preferred over calculating the odds ratio which is a poor estimate of the probability ratio when the prevalence of the outcomes is ≥10%.[94] In exploratory analyses, we will use interaction terms to examine whether label effects differ by participant socio-demographic characteristics. All analyses will use AmeriSpeak-provided sample weights to produce nationally representative estimates. To ensure transparency, reproducibility, and rigor, we will pre-register our analyses on both clinicaltrials.gov and aspredicted.org, and we will follow CONSORT guidelines.

5. *Answer the following* ***if they are relevant to your study design****:*

   A. *If the study has different arms, explain the process for assigning participants (intervention/control, case/control), including the sequence and timing of the assignment.*

Randomization. Participants will be randomized to one of five labeling conditions: 1) control (QR code) label, shown on all food items; 2) green 'low climate-impact' label, shown on all low climate-impact food items; 3) red 'high climate-impact' warning label, shown on all high-climate-impact food items; 4) 'climate-score' label, shown on all food items (one of five 'grades' will be shown for each labeled item'; and 5) traffic light label, with one of three levels shown on all food items.

   B. *If human biospecimens (blood, urine, saliva, etc.) will be collected, provide details about who will collect the specimen, the volume (ml) and frequency of collection, how the specimen will be used, stored, identified, and disposed of when the study is over. If specimens will be collected for use in future research (beyond this study), complete the "Biospecimen Repository" section below.*

   N/A

   C. *If genetic/genomic analyses are planned, address whether the data will be contributed to a GWAS or other large dataset. Address returning unanticipated incidental genetic findings to study participants.*

   N/A

   D. *If clinical or laboratory work will be performed at JHU/JHH, provide the JH Biosafety Registration Number.*

   N/A

   E. *If you will perform investigational or standard diagnostic laboratory tests using human samples or data, clarify whether the tests are validated and/or the lab is certified (for example is CLIA certified in the U.S.).* ***For clinical tests of human biospecimens, no results may be returned unless completed in a certified lab.*** *Explain the failure rate and under what*

circumstances you will repeat a test. For all human testing (biomedical, psychological, educational, etc.), clarify your plans for reporting test results to participants and/or to their families or clinicians. Address returning unanticipated incidental findings to study participants.

N/A

F. *If your study involves medical, pharmaceutical or other therapeutic intervention, provide the following information:*

a. *Will the study staff be blind to participant intervention status*?

No. Study staff will receive a deidentified dataset from NORC AmeriSpeak staff. The dataset will indicte the study arm the participant was randomized to.

b. *Will participants receive standard care or have current therapy stopped*?

N/A

c. *Will you use a placebo or non-treatment group, and is that justifiable*?

Yes, one arm of the study is a control group. This group will view the fast food menus without any climate impact labels. This is justifiable as this is the current status quo.

d. *Explain when you may remove a participant from the study*.

Participants may be removed for poor data quality (e.g., a high number of incomplete responses) as determined by NORC Amerispeak staff.

e. *What happens to participants on a study in which there is a medical intervention when the study ends? Will participants continue to have access to the study intervention? What happens if they leave the study early?*

N/A

f. *Describe the process for referring participants to care outside the study, if needed*.

N/A

VI. Data Custody, Management, Security, and Confidentiality Protections: *Data security and management plans must meet institutional standards. If you need assistance, contact [bsph_cybersecurity@jhu.edu]*

Investigators are responsible for ensuring the security of data from the time of collection, through any transfers from one system to another, analysis, sharing, storage, and ultimate archiving and disposal. The questions below seek to elicit your plans for these protections. Feel free to add information.

1. Data Sources: Identify the source(s) of data.

☒ Participant/Parent-Guardian/Legally Authorized Representative

☐ JHM Medical Records (from Epic)

Note for JHM Data Users Only: *Please complete the **Data Trust Risk Tiers Calculator** available on the Applications and Forms page on the JHSPH IRB website: [https://tinyurl.com/2p96md3s] and upload a copy of the documents to the "Miscellaneous- Other" section of your PHIRST application.*

In addition, review the ***Data Protection Attestation for Research and/or Healthcare Operations*** *at: [https://tinyurl.com/yszfkuur] and certify your attestation of compliance to those requirements.*

☐ **I certify my attestation of compliance to JHM Data Protection Requirements**

☐ Non-JHM Medical Records

☐ Outside Data Pprovider (CMS, National Death Index, Insurance Co., etc.)

☐ Other Existing Records (*please specify*):

2. Data Content: Will you collect, use, and/or record personal identifiers about study participants for any purpose? Please look at the list of identifiers in Question 3 to help answer this question. **Note: Limited Data Sets (including dates, ages, and zip codes) are considered to be "identifiable".**

☐ Yes: Continue with Question 3

☒ No: Skip to Question 6

3. Data Identification: Identify the Personally Identifiable Information (PII)/Protected Health Information (PHI) you will access/collect by checking the box(es) below for "Recruitment" and "Study Data" needs.

Recruitment	Study Data	PII/PHI to be Accessed/Collected
☐	☐	Name, signature, initials or other identifiable code
☐	☐	Geographic identifier (address, GPS location, etc.)
☐	☐	Dates (birth, death, clinical service, discharge, etc.)
☐	☐	Contact information (phone number, email address, etc.)
☐	☐	Identification numbers (SSN, driver's license, passport, etc.)
☐	☐	Health records identifiers (medical record #, insurance plan, etc.)
☐	☐	Text of clinical record notes
☐	☐	Device identifiers (implants, etc.)
☐	☐	Internet identifiers (IP address, social media accounts, etc.)
☐	☐	Biometric identifiers (fingerprints, retinal scan, voice print, etc.)
☐	☐	Audio Recordings
☐	☐	Video or full-face photographic images
☐	☐	Genomic / Genetic data
☐	☐	Other identifiers (*list here*):

4. Identifiers: If you have checked any of the boxes above, how will you protect personal identifiers?

☐ Will delete all identifiers (explain **when** you will delete identifiers):

☐ Will separate identifiers from analytic data and will store the link/code. Please explain where you will store the link/code:

☐ Will use a method to make it harder to connect the data with the study participant (jiggering date, use other methods to obfuscate, etc.). *Please explain:*

5. Data Transit Plans and Protections: Identifiable data may transfer, sometimes with multiple steps, from mechanisms for collection to storage. For example, participants may complete a web-based survey, which is then downloaded to a storage platform. Briefly identify these steps and the protections for each step (including encryption used at each step).

☐ Will delete all identifiers prior to transfer.

☐ Will separate identifiers from analytic data and will store the link/code prior to transfer. *Please explain where you will store link/code:*

☐ Other (*please specify*):

6. Device(s) used for <u>data collection</u>: Identify the computing device(s) being used for identifiable data receipt/collection. Check all that apply.

We understand that resources in low resource countries may require use of systems that are not pre-approved. The following are examples of platforms/storage solutions that are **<u>not pre-approved to store identifiable information</u>** and require a risk assessment from JHSPH Data Security. Do not hesitate to contact [*bsph_cybersecurity@jhu.edu*] for an assessment.

- JHU Independent Departmental Servers
- Local Computer owned by JH
- Other computers or devices owned/managed by study team members and used for other than secure web access
- USB/Portable data storage device
- Other solutions not managed by IT@JH, e.g., commercial cloud storage (Box, Dropbox, iCloud, personal OneDrive, Google Drive, Amazon storage, etc.)

☐ Provided or managed by JHSPH IT

☐ Study-provided, and not managed by JHSPH IT. These must include the following protective controls:
- Data encrypted while "at rest" (on a storage device)
- Security patches and updates are routinely or automatically applied
- Devices have access controls so that:
  - Each person accessing the device is uniquely identified (username)
  - Passwords are sufficiently strong to prevent compromise
  - All access is logged and recorded
  - Unauthorized access is prevented
- Approved access list is reviewed periodically for correctness

☒ Other (*please specify*): NORC AmeriSpeak will collect the data from their panel members via online survey.

7. Data Collection: Describe the format of data received/collected. Check all that apply.

☐ Paper/Hard Copy (must be secured in transit and placed in a secure cabinet/room)

☐ Audio recording

☐ Video recording

☐ Received directly by research team member and entered into file/database

☐ Mobile or Web App (custom developed). *Review* [*guidance*] *and provide attestation of compliance*

☐ Mobile or Web App (purchased). *Specify product and version*:

☒ Online survey. *Specify mechanism/platform*: NORC AmeriSpeak panel.

☐ 3rd party collector (*please specify*):

☐ Existing data shared with JHSPH by data provider via electronic access/transfer

☐ Duplicate and backup copies will be secured with same rigor as original data

☐ Other (*please specify*):

8. Devices/Platforms used for Analysis, Storage, Processing: Identify where the identifiable or de-identified data will be analyzed/stored. Check all that apply.

☐ Pre-approved storage and analysis platforms managed by JH/JHSPH for which security and risk mitigation measures are known.

Identify pre-approved storage platform(s) being used:

JHM Preferred:

☐ JH SAFE Desktop ☐ JH PMAP

Other Approved Platforms:

☒ JH One Drive/JHSPH OneDrive ☐ JH IT-Managed Network Storage ☐ JHM/JHSPH Qualtrics

☐ JHSPH HPCC ☐ JHSPH SharePoint ☐ JHSPH Shares ☐ JHU REDCap

☐ MARCC-Secure Environment

☐ Platform(s) not managed by JH/JHSPH, not pre-approved, and require a risk assessment review from JHSPH Data Security.

- Describe the not pre-approved platform(s) you plan to use:
- Describe the technologies you intend to use (software, hardware, connectivity) with a focus on the measures taken to secure collected data along the continuum of data collection, storage, transmittal and access:

9. Access to Data and Access Controls: How will you ensure that only authorized individuals can access the data? What access controls will you put into place to ensure that only authorized individuals may access and use the data. (For example, OneDrive [*guidance*] illustrates how to share files with "people you specify". [*BSPH Shares*] addresses providing permissions to individual people.) Check all that apply. Note: If you need assistance implementing secure access controls, contact [*bsph_cybersecurity@jhu.edu*]

☒ Will provide access to data in accordance with OneDrive/JHSPH-Shares guidance posted on JHU IT websites

☐ Will use secure access controls to limit access to individual-level data

☐ Will use secure access controls to provide other researchers controlled access only to aggregated study data

10. Data Sharing: Clarify if data are to be shared externally with third parties, including sponsors and other investigators, and whether only aggregated data will be shared, or if you will share individual-level data. Describe sharing and protection plans for that sharing, including the proposed use of data agreements.

Consider the following:

- *Information about your data sharing in the consent forms*
- *Information about data sharing laws in the country where data will be collected, and if they limit sharing, how you will comply with those limitations?*
- *Whether data will be shared in aggregate only, or individual level data*
- *Whether you plan to make the data publicly available, and in what form.*

☐ Will not share data with outside investigators

☐ Will make publicly available

☐ Will share with restrictions/controls

☐ Will share aggregated data only

☒ Will share individual-level data without identifiers

☐ Will deposit data into an existing data repository for future research. *Please explain*:

☐ Future research use and data sharing will have limited purposes. *Please explain*:

☐ Other sharing information:

11. Duration and Destruction: Explain how long data will be retained and the plan for eventual return, deidentification or destruction of data, including moving data to an archive.

We will retain data for a minimum of 7 years. All data is deidentified.

A. <u>Certificate of Confidentiality</u>:

All NIH studies include Certificate of Confidentiality (C of C) protections with the grant; the consent form must include the C of C language provided in our template. Other funders may obtain C of C protections through NIH. [https://grants.nih.gov/policy/humansubjects/coc.htm]

Does the study have Certificate of Confidentiality protections? Yes ☒ No ☐

VII. <u>Risks of the Study</u>:

A. Describe the risks, discomforts, and inconveniences associated with the study and its procedures, including physical, psychological, emotional, social, legal, or economic risks, and the risk of a breach of confidentiality. Include risks beyond individuals to include the study population as a group and community risks. <u>Ensure that the risks described in the consent documents are consistent with the risks outlined in the research plan</u>.

This study does not involve any major risks to study participants. The potential risks to participants are minimal. A potential risk could be a data breach, which is possible but very unlikely. The study team will never

have access to participant names or other identifying information. NORC AmeriSpeak, the survey research firm, will provide de-identified data to the research team when data collection is complete, so any data breach would be to de-identified data. Given this, and the data security procedures at NORC, the risk of a confidentiality or data breach is very low.

B. *Describe steps you will take to mitigate or minimize each of the risks described above. Include a description of your efforts to arrange for care or referral for participants who may need it.*

Study team members will not have any interaction with participants and will only receive deidentified data. We are not collecting any identifying information and will not be collecting IP Address information either. We will have no way of identifying the participant or their location.

C. *Describe the anticipated frequency and severity of the harms associated with the risks identified above; for example, if you are performing "x" test/assessment, or dispensing "y" drug, how often do you expect an "anticipated" adverse reaction to occur in a study participant, and how severe do you expect that reaction to be?*

D. *Describe the research burden for participants, including time, inconvenience, invasion of privacy in the home, out of pocket costs, etc*.

Participants will participant in one approximately 10 minute online survey at a time and place convenient to them.

E. *Describe how participant privacy, and if relevant – family privacy - will be protected during data collection if sensitive questions are included in interviews, or if study visits occur in the home setting.*

N/A

F. *Levels of COVID-19 community transmission will vary considerably by geography and over time, and therefore, the responses to the pandemic may also vary. The risk of COVID-19 to study staff and participants from in-person research activities can be mitigated by appropriate study procedures. If you are conducting in-person research activities, please indicate the protections you plan to implement at your research site(s):*

☒ Not applicable
☐ COVID testing of staff
☐ COVID testing of study participants
☐ Indoor masking/wearing PPE
☐ Social distancing for indoor activities
☐ Symptom screening of staff
☐ Symptom screening of study participants
☐ Vaccination of research team members
☐ Other procedures/comments:

VIII. <u>Direct Personal and Social Benefits</u>:

A. *Describe any potential direct benefits the study offers to participants ("payment" for participation is not a direct personal benefit).*

B. There are no immediate or direct benefits to study participants.

C. *Describe potential societal benefits likely to derive from the research, including value of knowledge learned.*

The benefits to society and the scientific community include greater understanding of the effectiveness of climate-impact menu labels in fast-food restaurants at promoting healthier and more sustainable dietary choices. Results will inform the design of effective labels- an area of growing industry and policy interest that could result in prevention of diet-related chronic diseases such as type-2 diabetes.

IX. Payment or Token of Appreciation:

A. *Do you plan to provide a non-monetary token of appreciation (food, soap, tea, chlorine tablets, etc.) to study participants? If no payment is provided, the JHSPH IRB strongly encourages providing such tokens. If yes, please describe below.*

No

B. *If you plan to provide a monetary payment, describe the form, amount, and schedule of payment to participants. Reimbursement for travel or other expenses is not "payment," and if the study will reimburse, explain*.

Participants are compensated by NORC AmeriSpeak according to their guidelines

C. *Include the possible total remuneration and any consequences for not completing all phases of the research.*

Level of compensation is set by the survey firm according to their guidelines. No consequences for not completing all phases (there is only 1 phase).

X. Study Management:

A. **Oversight Plan:**

1. *Describe how the study will be implemented. List all parties, including collaborators and subcontractors, who will be "engaged" in the human subjects research project and their roles .*

Dr. Julia Wolfson, Associate Professor in International Health, SPH, is the PI of the study and will lead data collection and analysis.

2. *What are the qualifications of study personnel implementing the project*?

Dr. Wolfson has a PhD in public health policy and 10 years experience conducting online survey research. The research team has extensive expertise in online experiments testing effects of food labels on food choices.

3. *How will non-professional personnel (data collectors) involved with the data collection and analysis be trained in human subjects research ethical protections? (Use the JHSPH Ethics Field Training Guide available on the JHSPH IRB website.* <u>If the study is a clinical trial, consider using the JHSPH Good Clinical Practice (GCP) For Social and Behavioral Research Field Guide).</u>

NORC AmeriSpeak staff will be involved in data collection. All staff are trained and certified in human subjects research.

4. *If the JHSPH PI is responsible for data collection and will not personally be on-site throughout the data collection process, provide details about PI site visits, the supervision over consent and data collection, and the communication plan between the PI and study team*.

   The PI will be in regular communication with NORC AmeriSpeak staff during study planning and data collection.

B. **Protocol Compliance and <u>Recordkeeping</u>:**

Describe how you plan to ensure that the study team follows the protocol and properly records and stores study data collection forms, IRB regulatory correspondence, and other study documentation (for assistance, contact: [*housecalls@jhu.edu*]).

<u>Please provide information about study oversight to ensure compliance with IRB approval and regulatory and institutional requirements.</u> If the study team does not follow study procedure, what is your plan for reporting protocol non-compliance?

Data, IRB correspondence, and other study documents will be stored on password protected computers.

C. **<u>Safety Monitoring:</u>**

1. *Describe how <u>participant safety</u> will be monitored as the study progresses, by whom, and how often. Will there be a medical monitor on site? If yes, who will serve in that role and what is that person's specific charge?*

   N/A

2. *If a Data Safety Monitoring Board (DSMB), or equivalent will be established, describe the following*:

   a. *The DSMB membership, affiliation and expertise*.

      N/A

   b. *The charge or charter to the DSMB*.

      N/A

   c. *Plans for providing DSMB reports to the IRB*.

      N/A

3. *Describe plans for interim analysis and stopping rules, if any*.

   N/A

D. **<u>Reporting Unanticipated Problems/Adverse Events (AEs) to the IRB (*all studies must complete this section*)</u>**:

*NOTE: The IRB does not require PROMPT reporting of all AEs, only those that are **<u>unanticipated, pose risk of harm to participants or others, and are related to the study</u>**. **Anticipated** AEs may be reported with the Continuing Review/Progress Report.*

Describe your plan for reporting to the JHSPH IRB, local IRBs, and (if applicable) to the sponsor. Include your plan for government-mandated reporting of child abuse or illegal activity.

We do not expect any adverse events, but should any occur we will report them immediately to the IRB.

E. **<u>Other IRBs/Ethics Review Boards</u>:**

If other IRBs will review the research, provide the name of each IRB/ethics review board and its Federal Wide Assurance number, if it has one (available on [*OHRP's Website*]*).* ***For federally funded studies, subrecipients MUST have a Federal Wide Assurance (FWA) number from the OHRP. The IRB overseeing the subrecipient should be registered with the OHRP. The JHSPH IRB will not have oversight responsibility for international subrecipients, and generally will not oversee data collection at external U.S. institutions Please contact the*** [*BSPH IRB Office*] ***with questions.***

Non-BSPH IRB/REC	FWA Number

F. **"Engaged" in Human Subjects Research:**

For studies that involve collaboration with non-JHSPH institutions, complete the chart below by describing the collaboration and the roles and responsibilities of each partner, including the JHSPH investigator. This information helps us determine what IRB oversight is required for each party. Complete the chart for all multi-collaborator studies.

Insert collaborator names and FWA numbers, if available. Note who will be "engaged" in human subjects research by filling in the following table:

	BSPH		
For federally funded studies, collaborators' FWA	00000287		
Primary Grant/Contract Recipient			
Grant/Contract Subrecipient			
Hiring Data Collectors			
Training Data Collectors			
Obtaining Informed Consent and/or Identifiable Data			
Accessing/Analyzing Identifiable Data			
Overseeing storage, access and use of biospecimens			

COMPLETE THE FOLLOWING SECTIONS WHEN RELEVANT TO YOUR STUDY:

XI. Secondary Data Analysis of Existing Data:

A. **Study Design**:

1. *Describe your study design and methods. The study design must relate to your stated aims/objectives.*

2. *Provide an estimated sample size and an explanation for that number.*

3. *Provide a brief data analysis plan and a description of variables to be derived.*

B. **Participants:**

1. *Describe the subjects who provided the original data and the population from which they were drawn.*

   Note: If you are receiving, accessing, or using data from a U.S. health care provider, the need for HIPAA review is likely. If you plan to bring identifiable health information from a foreign country to a U.S. covered entity (e.g., lab at the Hopkins SOM), HIPAA may be triggered. If either of these conditions is met, check "yes" to the HIPAA question in the PHIRST application.

2. *If you plan to analyze human specimens or genetic/genomic data, provide details about the source of those specimens and whether they were collected using an informed consent document. If yes, explain whether your proposed use is "consistent with" the scope of the original consent, if it potentially introduces new analyses beyond the scope of the original consent, and/or if it introduces new sensitive topics (HIV/STDs, mental health, addiction) or cultural/community issues that may be controversial.*

3. *Explain whether (and how) you plan to return results to the participants either individually or as a group*.

XII. Oversight Plan for Student-Initiated Studies:

A. For student-initiated studies, explain how the PI will monitor the student's adherence to the IRB-approved research plan, such as communication frequency and form, training, reporting requirements, and anticipated time frame for the research. Describe who will have direct oversight of the student for international studies if the PI will not personally be located at the study site, and their qualifications.

B. What is the data custody plan for student-initiated research? (Note: Students may not take identifiable information with them when they leave the institution.)

XIII. Creation of a Biospecimen Repository:

Explain the source of the biospecimens, if not described above, and what kinds of specimens will be retained over time. Clarify whether the specimens will be obtained specifically for repository purposes, or will be obtained as part of the core study and then retained in a repository.

A. Describe where the biospecimens will be stored and who will be responsible for them.

B. Describe how long the biospecimens will be stored, and what will happen at the end of that period.

C. Explain whether the biospecimens will be shared with other investigators, inside and outside of JHU, how the decision to share will be made, and by whom. Include your plans, if any, for commercial use. Also explain how downstream use of the specimen will be managed, and what will happen to left-over specimens.

D. *Describe whether future research using the biospecimens will include specimen derivation and processing (cell lines, DNA/RNA, etc.), genomic analyses, or any other work which could increase risk to participants. Explain what additional protections will be provided to participants.*

E. *If future research could yield unanticipated incidental findings (e.g., an unexpected finding with potential health importance that is not one of the aims of the study) for a participant, do you intend to disclose those findings to the study participant? Please explain your position.*

F. *Explain whether the specimens will be identifiable, and if so, how they will be coded, who will have access to the code, and whether the biospecimens will be shared in linked (identifiable) form*.

G. *Explain whether the repository will have Certificate of Confidentiality protections.*

H. *Explain whether a participant will be able to withdraw consent to use a biospecimen, and how the repository will handle a consent withdrawal request.*

I. *Describe data and/or specimen use agreements that will be required of users. Provide a copy of any usage agreement that you plan to execute with investigators who obtain biospecimens from you.*

XIV. Data Coordinating Center:

Complete if BSPH serves as the Data Coordinating Center.

A. *How will the study procedures be developed?*

B. *How will the study documents that require IRB approval at each local site be developed? Will there be some sort of steering or equivalent committee that will provide central review and approval of study documents, or will template consent forms, recruitment materials, data collection forms, etc. be developed by and provided to the local sites by the coordinating center without external review?*

C. *Will each local clinical site be overseen by its own IRB with an FWA, or will a Single IRB review the study? State whether the coordinating center will collect IRB approvals and renewals from the clinical centers; if not, explain why.*

D. *How will the coordinating center provide each local site with the most recent version of the protocol and other study documents? What will be the process for requesting that these updates be approved by local clinical center IRBs?*

E. *What is the plan for collecting data, managing the data, and protecting the data at the coordinating center?*

F. *What is the process for reporting and evaluating protocol events and deviations from the local sites? Who has overall responsibility for overseeing subject safety: the investigators at the recruitment site, the Coordinating Center, the Steering Committee, or a Data and Safety Monitoring Board (DSMB)? Is there a DSMB that will evaluate these reports and provide summaries of safety information to all the reviewing IRBs, including the coordinating center IRB? Please note that if there is a DSMB for the overall study, then the coordinating center PI does not have to report to the coordinating center IRB each individual adverse event/problem event that is submitted by the local site PIs.*

G. *Some FDA regulated studies have different AE reporting criteria than that required by the IRB (IRB Policy No. 103.06). How will you reconcile the different requirements, and who is responsible for this reconciliation?*

H. *Who is responsible for compliance with the study protocol and procedures and how will the compliance of the local sites be monitored and reviewed? How will issues with compliance be remedied?*

XV. Drug Products, Vitamins, Food and Dietary Supplements:

Complete this section if your study involves a drug, botanical, food, dietary supplement or other product that will be applied, inhaled, ingested or otherwise absorbed by the study participants. If you will be administering drugs, please upload the product information.

A. *List the name(s) of the study product(s), and the manufacturer/source of each product.*

Name of Study Product	Manufacturer/Source

B. *List each study product by name and indicate its approved/not approved status.*

Approved by the FDA and Commercially Available	Approved by Another Gov't Entity (provide name)	Cleared for Use at Local Study Site

C. *If your study product has an Investigational New Drug (IND) application through the U.S. Food and Drug Administration, provide the IND number, and the Investigators Brochure.*

Who will hold the IND?

D. *If your study product is a marketed drug, provide the package inserts or other product information. If the study product WILL NOT be used for its approved indication, dose, population, and route of administration, provide a detailed rationale justifying the off-label use of the study product.*

E. *If the study product does not require FDA approval (e.g., dietary supplements, botanicals, products not subject to the U.S. FDA, etc.), provide safety information (as applicable) and a certificate of analysis.*

F. *Explain who will be responsible for drug management and supply, labeling, dispensing, documentation and recordkeeping. Complete and upload into PHIRST the Drug Data Sheet available on the* [*BSPH IRB Website*]

G. *What drug monitoring and/or regulatory oversight will be provided as part of the study? Please describe.*

XVI. <u>Medical Devices:</u>

*Complete this section if your study will involve an approved or investigational medical device (**diagnostic**, non-significant risk, significant risk).*

A. *List the name(s) of the study product(s), the manufacturer/source of each product, and whether or not it is powered (electric, battery). Provide product information. If it is electric, upload documentation of clinical engineering approval or its equivalent from a local authority, to ensure that the device is in good working order.*

Name of Study Product	Manufacturer/Source	Powered?

B. *List each study product by name and indicate its status as approved by a government authority or not approved.*

Approved by the FDA and Commercially Available	Approved by Another Gov't Entity (provide name and approval information)	Not Approved

C. *If your investigational device is Exempt from the FDA IDE regulations, explain which section of the code applies to your device and why it meets the criteria provided. If it is a* ***diagnostic device****, provide pre-clinical information about the sensitivity and specificity of the test and the anticipated failure rate. If you plan to provide the results to participants or their physicians, justify doing so, and explain how those results will validated (or not) against the current "gold standard".*

D. *If you believe the investigational device is not IDE exempt under 21CFR 812.2(c), but is a "Non-Significant Risk" device considered to have an approved IDE application, provide information from the manufacturer supporting that position.*

E. *If you are using an investigational device that is a Significant Risk Device, provide the IDE number given by the FDA, <u>or if not under FDA jurisdiction</u>, explain why it is appropriate to use this device in this study. Provide a description of the device, and upload a picture or manufacturing schematics into PHIRST. Provide any other information relevant to a determination of its safety to be used for the purposes outlined in this research plan.*
