# Supplementary material for: Eco-Focused Menu Labels on Full Meal Orders From Fast-Food Restaurants: A Randomized Clinical Trial
Source: JAMA Health Forum. 2026 Jul 10;7(7):e262108. doi: 10.1001/jamahealthforum.2026.2108 (PMC13355149; doi:10.1001/jamahealthforum.2026.2108)
Supplement: Supplement 2. — eAppendix 1. Nutrition and Price Information Methods Appendix eAppendix 2. Greenhouse Gas Emissions Methods Appendix eAppendix 3. Results Appendix. eTable 1. Nutrition Profile Index, Calories, and Greenhouse Gas Emissions for Menu Items Shown to Participants eTable 2. Secondary Outcomes wording and response options eTable 3. Menu Item Selection Frequency (n [%]) by Experimental Condition (N=6210) eTable 4. Mean Predicted Nutrients of Full Meal Ordered by Experimental Condition and Restaurant (N=6210) eFigure 1. Mean Predicted Nutrition Profile Index Score of Meal Selected at the Burger Restaurant (Panel A) and the Sandwich Restaurant (Panel B) by Experimental Condition and Noticeability (n=6209) eTable 5. Mean Predicted Probabilities of Noticing Label, Identifying What Label was About, and Label Use by Treatment Condition eTable 6. Mean Predicted Probabilities of Correctly Identifying Higher Climate Impact Menu Items and Ranking Items by Climate Impact by Treatment Condition (n=6242) eFigure 2. Mean Predicted Nutrition Profile Index Score of Selected Main Item at the Burger Restaurant (Panel A) and the Sandwich Restaurant (Panel B) by Treatment Condition (n=6210) eFigure 3. Predicted Probability of Selecting Sustainable Item at the Burger Restaurant (Panel A) and the Sandwich Restaurant (Panel B) by Treatment Condition (n=6210) eFigure 4. Predicted Probability of Selecting Item Containing Red Meat the Burger Restaurant (Panel A) and the Sandwich Restaurant (Panel B) by Treatment Condition (n=6210) eFigure 5. Predicted Probability of Selecting a Sugar Sweetened Beverage at the Burger Restaurant (Panel A) and the Sandwich Restaurant (Panel B) by Treatment Condition (n=6210) eFigure 6. Mean Predicted Cost of selected Meals at the Burger Restaurant (Panel A) and the Sandwich Restaurant (Panel B) by Treatment Condition (n=6210) eTable 7. Summary Table of Key Results Across Label Conditions eAppendix 4. Menu images for different label conditions [file jamahealthforum-e262108-s002.pdf]

Supplemental Online Content

Wolfson JA, Tucker AC, Reimold AE, et al. Eco-focused menu labels on full meal orders from fast-food restaurants: a randomized clinical trial. *JAMA Health Forum.* Published online July 10, 2026. doi:10.1001/jamahealthforum.2026.2108

eAppendix 1. Nutrition and Price Information Methods Appendix

eAppendix 2. Greenhouse Gas Emissions Methods Appendix

eAppendix 3. Results Appendix.

eTable 1. Nutrition Profile Index, Calories, and Greenhouse Gas Emissions for Menu Items Shown to Participants

eTable 2. Secondary Outcomes wording and response options

eTable 3. Menu Item Selection Frequency (n [%]) by Experimental Condition (N=6210)

eTable 4. Mean Predicted Nutrients of Full Meal Ordered by Experimental Condition and Restaurant (N=6210)

eFigure 1. Mean Predicted Nutrition Profile Index Score of Meal Selected at the Burger Restaurant (Panel A) and the Sandwich Restaurant (Panel B) by Experimental Condition and Noticeability (n=6209)

eTable 5. Mean Predicted Probabilities of Noticing Label, Identifying What Label was About, and Label Use by Treatment Condition

eTable 6. Mean Predicted Probabilities of Correctly Identifying Higher Climate Impact Menu Items and Ranking Items by Climate Impact by Treatment Condition (n=6242)

eFigure 2. Mean Predicted Nutrition Profile Index Score of Selected Main Item at the Burger Restaurant (Panel A) and the Sandwich Restaurant (Panel B) by Treatment Condition (n=6210)

eFigure 3. Predicted Probability of Selecting Sustainable Item at the Burger Restaurant (Panel A) and the Sandwich Restaurant (Panel B) by Treatment Condition (n=6210)

© Wolfson JA 2026 et al. JAMA Health Forum.

eFigure 4. Predicted Probability of Selecting Item Containing Red Meat the Burger Restaurant (Panel A) and the Sandwich Restaurant (Panel B) by Treatment Condition (n=6210)

eFigure 5. Predicted Probability of Selecting a Sugar Sweetened Beverage at the Burger Restaurant (Panel A) and the Sandwich Restaurant (Panel B) by Treatment Condition (n=6210)

eFigure 6. Mean Predicted Cost of selected Meals at the Burger Restaurant (Panel A) and the Sandwich Restaurant (Panel B) by Treatment Condition (n=6210)

eTable 7. Summary Table of Key Results Across Label Conditions

eAppendix 4. Menu Images for different label conditions

This supplemental material has been provided by the authors to give readers additional information about their work.

© Wolfson JA 2026 et al. JAMA Health Forum.

"Effect of eco-focused menu labels on full meal orders from two fast-food restaurants: a nationally representative online randomized controlled trial"

APPENDIX

1. Nutrition and Price Information Methods Appendix
2. Greenhouse Gas Emissions Methods Appendix
3. Results Appendix.
    a. Appendix Tables and Figures presented in the order they appear in the manuscript.
4. Menu Images for different label conditions

© Wolfson JA 2026 et al. JAMA Health Forum.

1. Nutrition and Price Information Methods Appendix

The survey menus and menu items used in this study were modeled after Burger King and Subway. Nutrition information was primarily retrieved in August 2024 from each restaurant chain's website (Bk.com; Subway.com). Nutrition information was gathered directly from the restaurant's website for Burger King and from a document posted on the Subway's website dated April 2024.[1] Nutrition information retrieved included serving size, calories, total fat, saturated fat, trans fat, cholesterol, sodium, carbohydrates, fiber, sugar, and protein.

Serving sizes were not provided by Burger King in the nutrition information PDF nor in item descriptions on the restaurant's website. To retrieve serving size information, which was needed to calculate Nutrition Profile Index scores and used to estimate greenhouse gas emissions, supplemental nutrition information was retrieved from documents formerly on the Burger King's website dated April 2020 and November 2022,[2,3] and the 2021 update to MenuStat.org,[4] an interactive online database that contains historical nutrition information for a wide variety of US chain restaurants.

Nutrition information for packaged chips, fountain drinks, and bottled beverages on the Subway menu were collected from additional sources, because only calorie information was provided for those items by the restaurant. We matched items from Subway's website (in August 2024), with corresponding chips items on fastfoodfacts.org to retrieve package size and nutrition information. For fountain drinks, which were all Coca-Cola products, nutritional information from Coca-cola.com was used to calculate nutritional contents per fluid ounce of drink. The restaurant was contacted by phone to determine the volume of drink sizes offered and multiplied the per fluid ounce nutritional values by volume of each size. As a check, estimated calories from these calculations was compared against calories posted on the Subway website (referenced September 2024); the range of difference was between zero and 40 calories, with an average difference of ~12 calories. For bottled beverages, information for Pure Leaf Sweet Tea was gathered from Pepsico.com, and an assumption of no nutritional contents was made for Aquafina bottled water.

Prices displayed on survey menus beneath each food item were derived from real-world prices posted for different restaurant locations on the Burger King and Subway websites in January 2025. Prices were gathered for each restaurant from locations across 16 US cities of varying population size, including large metropolitan, metropolitan, and medium-sized urban cities (as defined by the Organization for Economic Cooperation and Development).[5-7] We selected four cities to sample restaurant prices from within each of four US Census regions: Northeast (New York, NY; Boston city, MA; Pittsburg, PA; Philadelphia, PA), Midwest (Cleveland city, OH; Indianapolis, IN; Detroit, MI; Chicago, IL), South (Houston, TX; Dallas, TX; Baltimore, MD; Raleigh city, NC) and West (Los Angeles, CA; Seattle, WA; Tucson, AZ; Aurora city, CO). Each individual price per item was determined by averaging its price across all 16 restaurant locations sampled. To align with Burger King and Subway pricing schemes, price averages per

© Wolfson JA 2026 et al. JAMA Health Forum.

item were rounded up to the nearest price ending with 9¢ (e.g., an average price of $7.85 increased to $7.89) for survey menu item prices.

References:

1. Subway. Subway. U.S. Nutrition Information. Subway. Accessed February 10, 2026. https://www.subway.com/en-us/-/media/northamerica/usa/nutrition/nutritiondocuments/us_nutrition_w3_4-16-2024.pdf
2. Burger King. Burger King USA Nuritionals: Core, Regional and Limited Time Offerings April 2020. Accessed February 2026, 2026. http://originqa.bk.com/pdfs/nutrition.pdf
3. Burger King. Burger King USA Nutrition Information. Accessed February 10, 2026. https://bk-use1-prod.sites.rbictg.com/nutrition/nutrition.pdf
4. The MenuStat Project. New York City Department of Health and Mental Hygiene. Accessed April 7, 2023. http://menustat.org/
5. Organisation for Economic Co-operation and Development. Urban Population by City Size. Accessed February 10, 2026. https://www.oecd.org/en/data/indicators/urban-population-by-city-size.html
6. United States Census Bureau. City and Town Population Totals: 2020-2024. Accessed February 10, 2026. https://www.census.gov/data/tables/time-series/demo/popest/2020s-total-cities-and-towns.html
7. United States Census Bureau. State Visualizations of Key Demographic Trends From the 2020 Census. Accessed February 10, 2026. https://www.census.gov/library/stories/state-by-state.html

© Wolfson JA 2026 et al. JAMA Health Forum.

2. Greenhouse Gas Emissions Methods Appendix

Estimation of menu item greenhouse gas emissions.

Greenhouse gas emissions (GHGE) per meal were calculated using data on the total carbon costs, including supply chain GHGE and carbon opportunity cost, in kg carbon dioxide equivalents (CO_2e) per kg food. Data were from the Coolfood Pledge Calculator and Coolfood Meals Calculator, developed by the World Resources Institute (WRI)[1].

The WRI GHGE data include 93 food items (e.g., red meat, poultry, cheese, tomatoes, potatoes) and account for GHGE from production sites (i.e., farms or fisheries) through to the point of purchase (including processing, transport, and packaging) and upstream supply chain losses, specific to North America. Feed crop production was included in the scope of GHGE for animal products. The carbon opportunity cost associated with each item was also included, i.e., the potential GHGE that could be sequestered if the land required for producing an item was reforested or rewilded. The carbon opportunity cost is based on the global average of past carbon losses.[1] Carbon dioxide equivalency was based on global warming potential over 100 years.

Each ingredient (e.g., bread, beef patty, lettuce) used in each menu item was matched to an item in the WRI GHGE data. In cases where a direct match was not available in WRI data, we selected the closest suitable proxy, e.g., jalapeno peppers were matched to WRI data for tomatoes on the rationale that both items are nightshade fruits. Beverages were excluded from GHGE calculations due to data limitations, and because the majority of beverage GHGE is attributed to packaging[2] which is a relatively small contributor to overall food supply chain GHGE.[3] Multiplying the mass of each ingredient by the associated GHGE per unit of mass yielded the $kgCO_2e$ associated with each ingredient. Summing the GHGE over all ingredients in a menu item yielded the total $kgCO_2e$ for the item. Note that while there were many assumptions and technical nuances involved in the calculations, the overwhelming share of GHGE was attributed to animal foods and thus decisions about other ingredients (e.g., bread, vegetables, condiments) in most cases had negligible effects on our results.

The aforementioned GHGE calculations required information about the specific ingredients in each menu item and the quantities of each ingredient. Ingredient lists were determined based on item names and descriptions available through the chain restaurant menu; however, the level of detail was, in many cases, limited to aggregate items (e.g., "lightly breaded chicken," "onion rings") that were themselves comprised of multiple ingredients (e.g., breading, oil, onion). Furthermore, ingredient quantities were not provided in many cases; exceptions included burgers, where the mass of the patty was often indicated in the description (e.g., "1/4 pound of beef"). To address these data gaps, we made the following assumptions: In cases where the quantity of an ingredient was not provided, we used standard serving sizes from the US Department of Agriculture (USDA) FoodData Central databases.[4] We also calculated the macronutrient content (i.e., protein, carbohydrates, fat) associated with each ingredient using USDA data or, where provided, nutrition information available via the restaurants. Comparing the total macronutrient content of the ingredients in a menu item against the macronutrient content reported by the restaurant menu allowed us to gauge whether our assumptions about ingredient amounts were defensible. For example, if the carbohydrate content we calculated for a sandwich item was lower than the carbohydrate content reported by the restaurant, we used this as an indication that our assumption about the serving size for bread was too low and thus we

© Wolfson JA 2026 et al. JAMA Health Forum.

adjusted amounts of the ingredient accordingly. Using this approach, we were able to parse out aggregate ingredients such onion rings into their approximate fractions of individual ingredients. Fat and carbohydrate content of onion rings provided by the restaurants, for example, provided clues about the relative amounts of oil and breading to include when modeling GHGE. The nutrient content of menu items, based on how we modeled them, were on average within ±5% of the macronutrient content reported by the restaurants, suggesting our estimates of ingredient mass, and menu item GHGE, were a close approximation.

The GHGE factors included in the WRI Calculators reflect retail weights of food (e.g., kg CO_2e per kg raw beef), whereas the weight of restaurant proteins was, in many cases, reported after some cooking losses occurred. Thus, for cooked proteins such as chicken and animal- and plant-based burgers, we divided the reported cooked mass of the protein by its cooking yield[5,6] to adjust the mass to retail weight. For burgers, we assumed the reported mass (e.g., ¼ pound) refers to the weight after pre-cooking but prior to on-site grilling, so we halved cooking losses for burgers on the assumption that some losses already occurred prior to arriving at the reported mass. For deli meats, we did not apply any cooking losses since the addition of byproducts, preservatives, and other fillers complicated efforts to estimate the retail mass of the original unprocessed meat.

Greenhouse gas emissions label designs.

We chose the labels in this study based on prior evidence regarding effective designs (e.g., traffic-light, warning labels) from the nutrition labeling literature,[7–11] and a prior study testing climate labels in a restaurant setting.[12] We also modeled the grade-scale label based on the already existing Eco-Score[13] and Nutri-Score labels,[14] and the numeric label on several numeric labels in use by the food industry.[15,16] We chose not to use a footprint icon after discussion within the research team about the appeal of showing a foot icon next to food items on a restaurant menu, and opted for a globe icon instead.

Greenhouse gas emissions label thresholds.

Thresholds for GHGE labels were set relative to the average regional meal-related GHGE in 2015 as the benchmark. Some thresholds also accounted for the necessary reductions in meal-related GHGE, by region, that would need to occur by 2050 to align with the climate mitigation goals set by the Paris Climate Agreement. The thresholds used for this study were calculated using the same methodology as described by Waite & Blondin (2022).[17] Specifically, global food-related GHGE need to decline in absolute terms by an estimated 67% between 2010 and 2050.[18] Because global food demand is projected to continue increasing by 56% during this period, the emissions intensity of agricultural production, or the GHGE per calorie, needs to fall even faster than absolute emissions.[18] Scaling these estimates to a 2015-2050 timeframe, GHGE would need to decrease by 59% while demand increases by 49%. A 72% reduction in global food-related emissions per calorie is thus needed per the following equation:

$$1-(1-0.59)/(1+0.49) = 0.72$$

The threshold between the "A" and "B" grade-scale labels was 1.7 kg CO_2e per meal (**Methods Appendix Table**). This represents a 72% relative reduction in emissions per calorie compared to the 2015 European average. This is the reduction in European food-related GHGE that would need to occur by 2050 to reduce the emissions intensity of food, in line with absolute reduction targets. This threshold represents a convergence target in which all regions converge

© Wolfson JA 2026 et al. JAMA Health Forum.

toward a single threshold. In the absence of a global target available in the methodology described by Waite & Blondin (2022), we selected the European target because per capita food system emissions in Europe were similar to per capita global emissions in 2015.[3] A convergence target is a more equitable and conservative global threshold in which the highest-consuming regions reduce emissions more relative to other regions.

The threshold between the “B” and “C” grade-scale labels and the green and yellow traffic-light labels was 2.6 kg CO_2e per meal. This represents a 72% relative reduction in 2015 North American per capita food-related GHGE, utilized because the study took place in North America. This threshold reflects a contraction target, in which emissions are reduced by at least 72% below the regional average diet (with all regions doing the same percent reduction). While it still represents an ambitious emissions reduction target, achieving it would mean that high-consuming regions such as North America would still have higher per-capita emissions than other regions of the world because they started from a higher baseline value.

The threshold between the “C” and “D” grade-scale labels, the yellow and red traffic-light labels, and the high-impact label was 9.2 kg CO_2e per meal. This is the estimated 2015 North American average per capita food-related GHGE per lunch or dinner, assuming 30% of emissions come from each of these meals.[17] Finally, the threshold between the “D” and “F” grade labels was 28 kg CO_2e per meal. This is three times greater than the average GHGE per meal in North America in 2015.

Methods Appendix Table: Greenhouse gas emissions label thresholds.

Label Design	Label Appearance	GHGE (kgCO_2e)
Traffic-Light	Green / Low-Impact	<2.6
	Yellow	2.6 - <9.2
	Red / High-Impact	≥9.2
Grade-Scale	A	<1.7
	B	1.7 - <2.6
	C	2.6 - <9.2
	D	9.2 - <27.6
	F	≥27.6

© Wolfson JA 2026 et al. JAMA Health Forum.

References

1. Waite R, Vennard D, Pozzi G. Tracking Progress Toward the Cool Food Pledge. Published online September 24, 2019. Accessed April 22, 2025. https://www.wri.org/research/tracking-progress-toward-cool-food-pledge
2. Amienyo D, Gujba H, Stichnothe H, Azapagic A. Life cycle environmental impacts of carbonated soft drinks. *Int J Life Cycle Assess*. 2013;18(1):77-92. doi:10.1007/s11367-012-0459-y
3. Crippa M, Solazzo E, Guizzardi D, Monforti-Ferrario F, Tubiello FN, Leip A. Food systems are responsible for a third of global anthropogenic GHG emissions. *Nat Food*. 2021;2(3):198-209. doi:10.1038/s43016-021-00225-9
4. USDA FoodData Central. Accessed May 5, 2025. https://fdc.nal.usda.gov/
5. Zhou H, Vu G, Gong X, McClements DJ. Comparison of the Cooking Behaviors of Meat and Plant-Based Meat Analogues: Appearance, Texture, and Fluid Holding Properties. *ACS Food Sci Technol*. 2022;2(5):844-851. doi:10.1021/acsfoodscitech.2c00016
6. *USDA Table of Cooking Yields for Meat and Poultry*. U.S. Department of Agriculture, Agricultural Research Service; 2012. Accessed May 5, 2025. Nutrient Data Laboratory Home Page: http://www.ars.usda.gov/nutrientdata
7. Front-of-package labeling. Global Food Research Program. Accessed April 23, 2025. https://www.globalfoodresearchprogram.org/resource/front-of-package-label-maps/
8. Grummon AH, Reimold AE, Hall MG. Influence of the San Francisco, CA, Sugar-Sweetened Beverage Health Warning on Consumer Reactions: Implications for Equity from a Randomized Experiment. *J Acad Nutr Diet*. 2022;122(2):363-370.e6. doi:10.1016/j.jand.2021.07.008
9. Roberto CA, Ng SW, Ganderats-Fuentes M, et al. The Influence of Front-of-Package Nutrition Labeling on Consumer Behavior and Product Reformulation. *Annu Rev Nutr*. 2021;41(Volume 41, 2021):529-550. doi:10.1146/annurev-nutr-111120-094932
10. Grummon AH, Hall MG. Sugary drink warnings: A meta-analysis of experimental studies. *PLOS Med*. 2020;17(5):e1003120. doi:10.1371/journal.pmed.1003120
11. Grummon AH, Gibson LA, Musicus AA, Stephens-Shields AJ, Hua SV, Roberto CA. Effects of 4 Interpretive Front-of-Package Labeling Systems on Hypothetical Beverage and Snack Selections: A Randomized Clinical Trial. *JAMA Netw Open*. 2023;6(9):e2333515. doi:10.1001/jamanetworkopen.2023.33515
12. Wolfson JA, Musicus AA, Leung CW, Gearhardt AN, Falbe J. Effect of Climate Change Impact Menu Labels on Fast Food Ordering Choices Among US Adults: A Randomized Clinical Trial. *JAMA Netw Open*. 2022;5(12):e2248320. doi:10.1001/jamanetworkopen.2022.48320
13. Taillie LS, Wolfson JA, Prestemon CE, et al. The impact of an eco-score label on US consumers' perceptions of environmental sustainability and intentions to purchase food: A randomized experiment. Gill AR, ed. *PLOS ONE*. 2024;19(6):e0306123. doi:10.1371/journal.pone.0306123

© Wolfson JA 2026 et al. JAMA Health Forum.

14. Julia C, Hercberg S. Development of a new front-of-pack nutrition label in France: the five-colour Nutri-Score. *Public Health Panor*. 2017;3(4):712-725.

15. Carbon Labeling | Just Salad. Accessed February 18, 2025. https://www.justsalad.com/carbonlabel

16. Chipotle Sets Science Based Climate Goals To Reduce Its Carbon Emissions 50% By 2030 - Nov 4, 2021. Accessed February 18, 2025. https://newsroom.chipotle.com/2021-11-04-Chipotle-Sets-Science-Based-Climate-Goals-To-Reduce-Its-Carbon-Emissions-50-By-2030

17. Waite R, Blondin S. Identifying Cool Food Meals. Published online June 27, 2022. Accessed May 19, 2025. https://www.wri.org/research/identifying-cool-food-meals

18. Searchinger T, Waite R, Hanson J, Ranganathan P, Dumas P, Matthews E. *World Resources Report: Creating a Sustainable Food Future—A Menu of Solutions to Feed Nearly 10 Billion People by 2050 (Final Report).* World Resources Institute; 2019. Accessed May 24, 2025. http://www. sustainablefoodfuture.org

© Wolfson JA 2026 et al. JAMA Health Forum.

3. **Results Appendix**

Table e1. Nutrition Profile Index, Calories, and Greenhouse Gas Emissions for Menu Items Shown to Participants

Item	NPI Score	Calories	Healthy (NPI score >64)	GHGE	Sustainable (GHGE<2.6)
**Burger Restaurant**					
Whopper	48	670	No	25.45	No
Whopper with Cheese	46	770	No	26.3	No
Whopper with Bacon and Cheese	42	820	No	27.84	No
Impossible Whopper	52	630	No	1.68	Yes
Double Whopper	48	920	No	49.9	No
Double Whopper with Cheese	44	1040	No	51.59	No
Triple Whopper	48	1170	No	74.34	No
Triple Whopper with Cheese	44	1300	No	75.19	No
Bacon King	34	1200	No	51.89	No
Bacon Double Cheeseburger	38	440	No	21.58	No
Bacon Cheeseburger	38	340	No	11.38	No
Double Cheeseburger	44	400	No	20.81	No
Cheeseburger	40	290	No	10.61	No
Hamburger	46	250	No	10.19	No
Royal Crispy Chicken Sandwich	48	600	No	3.38	No
Fiery Royal Crispy Chicken Sandwich	50	690	No	3.41	No
Bacon Swiss Crispy Chicken Sandwich	42	740	No	5.77	No
Chicken Sandwich	48	680	No	2.25	Yes
8pc Nuggets	44	390	No	1.78	Yes
Big Fish	50	570	No	2.47	Yes
**Sandwich Restaurant**					
Black Forest Ham 6	74	280	Yes	2.49	Yes
Black Forest Ham Footlong	74	560	Yes	4.98	No
B.L.T 6	64	370	Yes	3.21	No

© Wolfson JA 2026 et al. JAMA Health Forum.

B.L.T Footlong	64	740	Yes	6.43	No
Cold Cut Combo 6	66	330	Yes	1.95	Yes
Cold Cut Combo Footlong	66	660	Yes	3.9	No
Grilled Chicken 6	76	300	Yes	2.61	No
Grilled Chicken Footlong	76	600	Yes	5.22	No
Meatball Marinara 6	62	460	No	49	No
Meatball Marinara Footlong	62	920	No	98.01	No
Oven-Roasted Turkey 6	74	270	Yes	1.81	Yes
Oven-Roasted Turkey Footlong	74	540	Yes	3.63	No
Roast Beef 6	74	310	Yes	17.98	No
Roast Beef Footlong	74	620	Yes	35.96	No
Steak & Cheese 6	62	370	No	23.36	No
Steak & Cheese Footlong	62	740	No	46.72	No
Tuna 6	68	480	Yes	2.07	Yes
Tuna Footlong	68	960	Yes	4.15	No
Veggie Delite 6	76	220	Yes	0.36	Yes
Veggie Delite Footlong	76	440	Yes	0.71	Yes
Oven-Roasted Turkey Wrap	70	410	Yes	2.6	Yes
Steak & Cheese Wrap	62	560	No	46.55	No
Tuna Wrap	66	800	Yes	3.11	No
Grilled Chicken Wrap	76	460	Yes	4.19	No
Veggie Delite Wrap	76	310	Yes	0.54	Yes
Black Forest Ham Wrap	70	430	Yes	3.95	No
Cold Cut Combo Wrap	64	520	Yes	2.86	No
Roast Beef Wrap	72	480	Yes	34.93	No
Oven-Roasted Turkey Salad	84	110	Yes	1.45	Yes
Steak & Cheese Salad	76	210	Yes	23.47	No
Tuna Salad	80	310	Yes	1.71	Yes
Grilled Chicken Salad	88	130	Yes	2.25	Yes
Veggie Delite Salad	82	50	Yes	0.36	Yes
Black Forest Ham Salad	84	120	Yes	2.13	Yes

© Wolfson JA 2026 et al. JAMA Health Forum.

Cold Cut Combo Salad	82	160	Yes	1.59	Yes
Roast Beef Salad	84	150	Yes	17.62	No
Meatball Marinara Salad	74	300	Yes	48.66	No

© Wolfson JA 2026 et al. JAMA Health Forum.

Table e2. Secondary Outcomes wording and response options

Survey item	Item wording	Response options	Reference/ Notes
Greenhouse Gas Emissions of selected meals	N/A	Continuous measure of total kilograms of carbon dioxide equivalent (kgCO2e) summed across each menu item selected.	See methods appendix for more detail.
Perceptions of labeled items (looped over 4 items/ restaurant)	For the next set of questions, please view each menu item and answer the following questions about it.		Asked in random order for 4 items/ restaurant (order also randomized).
	How appealing do you think this item is?	1. Very unappealing<br>2. Unappealing<br>3. Slightly unappealing<br>4. Neither appealing nor unappealing<br>5. Slightly appealing<br>6. Appealing<br>7. Very appealing	
	How healthy do you think this item is?	1. Very unhealthy<br>2. Unhealthy<br>3. Slightly unhealthy<br>4. Neither unhealthy nor healthy<br>5. Slightly healthy<br>6. Healthy<br>7. Very healthy	
	What do you think the climate impact of this item is?	1. Extremely high climate impact<br>2. Very high climate impact<br>3. High climate impact	

© Wolfson JA 2026 et al. JAMA Health Forum.

		4. Moderate climate impact<br>5. Low climate impact<br>6. Very low climate impact<br>7. Extremely low climate impact	
Label noticing	On the menu you just saw, did you notice any labels (other than calories) next to or below the menu items?	1. Yes<br>2. No<br>77. I don't know/ I can't remember	
Label noticing (2) if "yes" to above	What did the labels you saw next to or below the menu items tell you about?	1. Sugar<br>2. Sodium<br>3. QR code<br>4. Climate impact<br>5. Organic<br>6. Healthy<br>7. Unhealthy<br>8. None of these<br>77. I don't know/ I can't remember	
Label use	Did you use the labels you saw when deciding what to order?	1. Yes<br>2. No	
Perceived Message Effectiveness (PME) intro	Considering this label [participants' assigned label], how much do you agree with the following statements:		Adapted from: Baig SA, Noar SM, Gottfredson NC, Boynton MH, Ribisl KM, Brewer NT. UNC Perceived Message Effectiveness: Validation of a Brief Scale. *Ann Behav Med.* 2019;53(8):732-742. doi:10.1093/abm/kay080

© Wolfson JA 2026 et al. JAMA Health Forum.

PME discourage	This label discourages me from wanting to eat foods with a high climate impact.	Not at all<br><br>A little bit<br><br>Somewhat<br><br>Quite a bit<br><br>A great deal	Adapted from: Baig SA, Noar SM, Gottfredson NC, Boynton MH, Ribisl KM, Brewer NT. UNC Perceived Message Effectiveness: Validation of a Brief Scale. *Ann Behav Med*. 2019;53(8):732-742. doi:10.1093/abm/kay080
PME unpleasant	This label makes consuming menu items with high climate impact seem unpleasant.	Not at all<br><br>A little bit<br><br>Somewhat<br><br>Quite a bit<br><br>A great deal	Adapted from: Baig SA, Noar SM, Gottfredson NC, Boynton MH, Ribisl KM, Brewer NT. UNC Perceived Message Effectiveness: Validation of a Brief Scale. *Ann Behav Med*. 2019;53(8):732-742. doi:10.1093/abm/kay080
PME concerned-health effects	This label makes me concerned about the health effects of eating menu items with a high climate impact.	Not at all<br><br>A little bit<br><br>Somewhat<br><br>Quite a bit<br><br>A great deal	Adapted from: Baig SA, Noar SM, Gottfredson NC, Boynton MH, Ribisl KM, Brewer NT. UNC Perceived Message Effectiveness: Validation of a Brief Scale. *Ann Behav Med*. 2019;53(8):732-742. doi:10.1093/abm/kay080
PME concerned-environmental effects	This label makes me concerned about the environmental effects of eating menu items with a high climate impact.	Not at all<br><br>A little bit<br><br>Somewhat<br><br>Quite a bit<br><br>A great deal	Adapted from: Baig SA, Noar SM, Gottfredson NC, Boynton MH, Ribisl KM, Brewer NT. UNC Perceived Message Effectiveness: Validation of a Brief Scale. *Ann Behav Med*. 2019;53(8):732-742. doi:10.1093/abm/kay080

© Wolfson JA 2026 et al. JAMA Health Forum.

Believability	This label is believable to me.	Not at all<br><br>A little bit<br><br>Somewhat<br><br>Quite a bit<br><br>A great deal	
Attention	This label grabs my attention.	Not at all<br><br>A little bit<br><br>Somewhat<br><br>Quite a bit<br><br>A great deal	
Knowledge of climate impact	Please rank the below food options from smallest (1) to largest (3) climate impact.	A. Meal in which the main protein is beef.<br>B. Meal in which the main protein is chicken.<br>C. Meal in which the main protein is tofu.	
	Which menu item below has a larger contribution to climate change (i.e., produces more greenhouse gas emissions)?	1. Image of item 1<br>2. Image of item 2<br>3. The menu items have the same climate impact	Asked twice in random order, comparing 2 items/ restaurant
Nutrient Profile Index (NPI) score of main item	N/A	Measured with a 0 to 100-point score for foods; a score of >=64 is considered healthy. NPI awards points from nutrients to encourage (e.g., fiber, protein) and nutrients of concern	Based on the United Kingdom (UK) Ofcom Nutrient Profiling Model. See methods for more detail.

© Wolfson JA 2026 et al. JAMA Health Forum.

		(e.g., sodium, sugar) per 100 grams.	
Red meat selection	N/A	Measured as a dichotomous variable (yes/no) indicating whether participants' hypothetical meal orders include an item containing red meat.	
Sugar-sweetened beverage selection	N/A	Measured as a dichotomous variable (yes/no) indicating whether participants' hypothetical meal orders include a sugar-sweetened beverage.	
Total energy selected	N/A	The total number of calories in participants' hypothetical meal orders, calculated as the sum of calories across all menu items selected.	
Total amount of saturated fat selected	N/A	The total amount of saturated fat in participants' hypothetical meal orders, calculated as the sum of saturated fat (grams) across all menu items selected.	
Total amount of sugar selected	N/A	The total amount of sugar in participants' hypothetical meal orders, calculated as the sum of sugar (grams) across all menu items selected.	
Total amount of protein selected	N/A	The total amount of sugar in participants' hypothetical meal orders, calculated as the sum of sugar (grams) across all menu items selected.	
Total amount of fiber selected	N/A	The total amount of fiber in participants'	

© Wolfson JA 2026 et al. JAMA Health Forum.

		hypothetical meal orders, calculated as the sum of fiber (grams) across all menu items selected.	
Total amount of sodium selected	N/A	The total amount of sodium in participants' hypothetical meal orders, calculated as the sum of sodium (milligrams) across all menu items selected.	
Total price of meal order	N/A	Measured by the cumulative total price (USD) of participants' hypothetical meal orders per restaurant, based on the prices displayed on the survey menus.	

Table e3. Menu Item Selection Frequency (n [%]) by Experimental Condition (N=6210)

Item	Overall (n=6210)	Control (n=1236)	Low-Impact (n=1249)	Traffic-Light (n=1235)	High-Impact (n=1243)	Grade-Scale (n=1247)	p-value
**Burger Restaurant**							
Whopper	551 (9.1)	129 (11.0)	122 (10.0)	88 (6.5)	101 (7.9)	111 (9.8)	0.03
Whopper with Cheese	644 (10.5)	120 (10.4)	139 (10.8)	124 (11.2)	130 (9.2)	131 (10.8)	0.76
Whopper with Bacon and Cheese	474 (7.6)	102 (7.9)	101 (8.0)	82 (6.9)	95 (8.0)	94 (7.4)	0.91
Impossible Whopper	452 (6.7)	50 (3.4)	100 (7.2)	118 (9.8)	78 (5.6)	106 (7.8)	**<0.001**
Double Whopper	128 (1.9)	27 (2.2)	27 (1.8)	20 (1.5)	28 (2.1)	26 (1.9)	0.84
Double Whopper with Cheese	273 (4.8)	59 (5.2)	65 (6.0)	37 (2.6)	56 (6.3)	56 (3.8)	0.01
Triple Whopper	47 (0.8)	7 (0.4)	13 (1.3)	13 (1.2)	5 (0.6)	9 (0.6)	0.22
Triple Whopper with Cheese	147 (2.5)	29 (2.7)	33 (2.5)	26 (2.0)	28 (3.1)	31 (2.4)	0.78
Bacon King	285 (4.7)	57 (4.0)	53 (3.7)	47 (3.9)	64 (6.2)	64 (5.5)	0.12
Bacon Double Cheeseburger	226 (4.0)	66 (5.0)	46 (4.3)	36 (3.7)	43 (3.3)	35 (3.7)	0.51
Bacon Cheeseburger	180 (2.7)	46 (3.7)	40 (3.0)	18 (1.2)	31 (2.1)	45 (3.3)	0.03
Double Cheeseburger	180 (3.1)	39 (3.5)	28 (1.5)	41 (4.2)	39 (2.9)	33 (3.5)	0.09
Cheeseburger	174 (2.7)	49 (4.1)	35 (2.4)	25 (2.1)	34 (2.6)	31 (2.4)	0.10
Hamburger	123 (1.9)	27 (2.0)	24 (2.4)	22 (1.4)	24 (2.0)	26 (1.7)	0.70
Royal Crispy Chicken Sandwich	361 (5.7)	67 (5.0)	72 (6.1)	78 (6.1)	86 (6.8)	58 (4.5)	0.39
Fiery Royal Crispy Chicken Sandwich	333 (5.0)	75 (5.7)	48 (3.5)	67 (5.4)	81 (5.5)	62 (5.1)	0.25
Bacon Swiss Crispy Chicken Sandwich	342 (5.3)	61 (5.0)	52 (4.4)	91 (6.5)	79 (5.4)	59 (5.3)	0.53
Chicken Sandwich	484 (7.9)	78 (7.5)	85 (6.6)	123 (9.2)	94 (7.7)	104 (8.3)	0.49
8pc Nuggets	302 (5.0)	54 (4.7)	69 (5.7)	69 (5.9)	53 (5.1)	57 (3.8)	0.42
Big Fish	504 (7.9)	94 (6.5)	97 (8.7)	110 (8.5)	94 (7.7)	109 (8.3)	0.57
**Sandwich Restaurant**							
Black Forest Ham 6	295 (4.9)	47 (3.6)	60 (5.4)	75 (6.7)	57 (4.3)	56 (4.7)	0.09
Black Forest Ham Footlong	303 (4.6)	56 (4.3)	62 (4.4)	63 (4.6)	64 (4.6)	58 (4.9)	0.99
B.L.T 6	144 (2.3)	32 (2.5)	22 (1.8)	28 (2.5)	30 (2.2)	32 (2.5)	0.87
B.L.T Footlong	226 (3.4)	39 (3.0)	36 (2.3)	58 (5.1)	48 (3.3)	45 (3.6)	0.03
Cold Cut Combo 6	261 (4.3)	53 (4.4)	47 (3.2)	48 (4.4)	53 (4.9)	60 (4.8)	0.55

© Wolfson JA 2026 et al. JAMA Health Forum.

Cold Cut Combo Footlong	404 (6.4)	71 (5.1)	76 (6.8)	84 (6.9)	98 (7.1)	75 (6.0)	0.52
Grilled Chicken 6	202 (3.0)	48 (3.8)	34 (2.9)	32 (2.4)	51 (3.3)	37 (2.8)	0.53
Grilled Chicken Footlong	352 (5.5)	72 (5.2)	68 (5.8)	71 (6.3)	71 (4.4)	70 (6.0)	0.52
Meatball Marinara 6	145 (2.4)	35 (2.9)	36 (3.2)	21 (2.0)	27 (2.0)	26 (1.7)	0.20
Meatball Marinara Footlong	274 (5.0)	64 (6.1)	68 (5.5)	45 (3.6)	47 (6.0)	50 (3.7)	0.08
Oven-Roasted Turkey 6	543 (8.6)	96 (8.2)	111 (8.1)	138 (12.0)	97 (7.6)	101 (7.4)	0.01
Oven-Roasted Turkey Footlong	429 (7.2)	96 (8.4)	77 (6.0)	78 (6.0)	103 (8.9)	75 (6.8)	0.11
Roast Beef 6	83 (1.2)	22 (1.6)	19 (1.3)	10 (0.5)	13 (1.2)	19 (1.3)	0.21
Roast Beef Footlong	145 (2.5)	31 (2.0)	38 (3.5)	27 (2.4)	22 (2.2)	27 (2.4)	0.43
Steak & Cheese 6	202 (3.7)	56 (4.8)	50 (4.5)	27 (2.3)	27 (2.5)	42 (4.6)	0.04
Steak & Cheese Footlong	599 (10.1)	135 (11.7)	129 (10.2)	86 (7.1)	120 (10.9)	129 (10.4)	0.06
Tuna 6	230 (3.4)	29 (2.7)	49 (3.8)	56 (4.1)	43 (2.6)	53 (3.9)	0.29
Tuna Footlong	197 (3.1)	42 (3.8)	31 (2.6)	46 (3.5)	52 (4.0)	26 (1.4)	0.03
Veggie Delite 6	117 (2.0)	12 (1.0)	27 (1.7)	28 (2.5)	20 (2.0)	30 (3.1)	0.09
Veggie Delite Footlong	145 (2.3)	28 (2.3)	36 (3.2)	23 (1.9)	22 (1.3)	36 (2.6)	0.14
Oven-Roasted Turkey Wrap	126 (1.8)	14 (0.8)	25 (2.0)	36 (2.4)	21 (2.0)	30 (2.0)	0.15
Steak & Cheese Wrap	71 (1.0)	20 (1.3)	13 (1.1)	9 (0.6)	14 (0.8)	15 (1.4)	0.44
Tuna Wrap	34 (0.6)	4 (0.7)	7 (0.5)	7 (0.7)	10 (0.7)	6 (0.5)	0.92
Grilled Chicken Wrap	148 (2.2)	33 (2.4)	19 (1.5)	24 (1.4)	40 (2.7)	32 (3.0)	0.13
Veggie Delite Wrap	47 (0.8)	9 (0.4)	12 (0.9)	11 (1.0)	6 (0.4)	9 (1.2)	0.21
Black Forest Ham Wrap	23 (0.5)	7 (0.6)	4 (0.1)	7 (0.6)	2 (0.5)	3 (0.8)	0.62
Cold Cut Combo Wrap	23 (0.4)	4 (0.4)	7 (0.6)	3 (0.2)	6 (0.6)	3 (0.3)	0.45
Roast Beef Wrap	19 (0.2)	5 (0.2)	2 (0.2)	6 (0.3)	1 (0.2)	5 (0.3)	0.97
Oven-Roasted Turkey Salad	83 (1.2)	16 (1.1)	17 (1.7)	12 (0.8)	15 (1.1)	23 (1.2)	0.52
Steak & Cheese Salad	42 (0.7)	12 (0.8)	8 (0.6)	4 (0.5)	3 (0.1)	15 (1.1)	0.09
Tuna Salad	56 (0.9)	8 (0.5)	11 (1.1)	14 (1.1)	11 (0.9)	12 (0.8)	0.70
Grilled Chicken Salad	146 (2.2)	23 (1.7)	28 (2.1)	36 (2.4)	33 (3.1)	26 (1.6)	0.28
Veggie Delite Salad	24 (0.3)	5 (0.3)	4 (0.2)	3 (0.1)	4 (0.3)	8 (0.4)	0.71
Black Forest Ham Salad	26 (0.4)	3 (0.2)	8 (0.6)	8 (0.3)	4 (0.7)	3 (0.1)	0.20

© Wolfson JA 2026 et al. JAMA Health Forum.

Cold Cut Combo Salad	29 (0.6)	4 (0.5)	5 (0.5)	7 (0.6)	4 (0.4)	9 (1.0)	0.62
Roast Beef Salad	10 (0.1)	3 (0.3)	1 (0.0)	3 (0.1)	2 (0.2)	1 (0.0)	0.14
Meatball Marinara Salad	7 (0.1)	2 (0.3)	2 (0.1)	1 (0.1)	2 (0.1)	0	0.49

Note: Statistical significance at <0.05 with Bonferroni-Holm correction for multiple comparisons within family of outcomes. Bolded scores remained significant after Bonferroni-Holm correction for multiple comparisons.

© Wolfson JA 2026 et al. JAMA Health Forum.

Table e4. Mean Predicted Nutrients of Full Meal Ordered by Experimental Condition and Restaurant (N=6210)

	Control (n= 1260)	Low-Impact (n=1262)		Traffic-Light (n=1252)		High-Impact (n=1260)		Grade-Scale (n=1260)	
	Mean (95% CI)	Mean (95% CI)	p-value	Mean (95% CI)	p-value	Mean (95% CI)	p-value	Mean (95% CI)	p-value
Burger Restaurant									
Total Calories[a]	1366 (1332, 1401)	1339 (1304, 1374)	0.27	1345 (1315, 1376)	0.38	1387 (1350, 1425)	0.42	1363 (1329, 1397)	0.90
Protein[a]	40.4 (39.2, 41.6)	40.5 (39.2, 41.7)	0.95	39.7 (38.7, 40.7)	0.37	42.0 (40.6, 43.3)	0.09	40.7 (39.6, 41.9)	0.70
Total Fiber[a]	8.0 (7.8, 8.2)	7.9 (7.7, 8.1)	0.52	8.2 (8.0, 8.4)	0.17	8.1 (7.9, 8.3)	0.74	8.2 (8.0, 8.5)	0.17
Total Sugar[b]	71.8 (68.1, 75.5)	68.4 (64.7, 72.0)	0.20	67.9 (64.2, 71.7)	0.15	71.8 (67.7, 75.9)	0.99	67.4 (63.7, 71.1)	0.10
Saturated Fat[a]	19.6 (18.9, 20.3)	19.3 (18.5, 20.1)	0.56	18.9 (18.2, 19.6)	0.15	20.0 (19.2, 20.8)	0.49	19.7 (18.9, 20.5)	0.82
Sodium[a]	1982 (1939, 2025)	1956 (1915, 1997)	0.40	1990 (1953, 2027)	0.78	2017 (1971, 2063)	0.27	2005 (1963, 2047)	0.45
Sandwich Restaurant									
Total Calories[a]	984 (958, 1011)	957 (930, 983)	0.15	966 (939, 993)	0.34	983 (954, 1011)	0.93	940 (914, 966)	0.02
Protein[a]	37.4 (36.5, 38.4)	36.4 (35.3, 37.4)	0.14	35.3 (34.3, 36.3)	0.003	37.1 (36.2, 38.1)	0.70	36.1 (35.1, 37.2)	0.07
Total Fiber[a]	7.7 (7.5, 8.0)	7.7 (7.5, 7.9)	0.81	7.7 (7.5, 7.9)	0.71	7.8 (7.6, 8.0)	0.83	7.5 (7.3, 7.7)	0.08
Total Sugar[b]	56.4 (52.9, 59.9)	55.3 (51.8, 58.8)	0.61	59.2 (55.6, 62.8)	0.28	58.7 (55.0, 62.3)	0.38	52.7 (49.3, 56.1)	0.14
Saturated Fat[a]	9.7 (9.3, 10.1)	9.4 (9.0, 9.8)	0.31	9.2 (8.8, 9.6)	0.08	9.6 (9.1, 10.0)	0.67	9.2 (8.9, 9.6)	0.07
Sodium[c]	1748 (1695, 1801)	1693 (1639, 1747)	0.40	1656 (1605, 1706)	0.90	1756 (1700, 1812)	0.36	1653 (1604, 1702)	0.07

Note: Statistical significance at <0.05 with Bonferroni-Holm correction for multiple comparisons within family of outcomes. Post estimation margins after weighted simple linear regression

a. Post estimation margins after weighted generalized linear model with a gamma family and log link
b. Post estimation margins after weighted simple linear regression with interaction term for the order restaurant menus were presented.

© Wolfson JA 2026 et al. JAMA Health Forum.

Figure e1. Mean Predicted Nutrition Profile Index Score of Meal Selected at the Burger Restaurant (Panel A) and the Sandwich Restaurant (Panel B) by Experimental Condition and Noticeability (n=6209) [a]

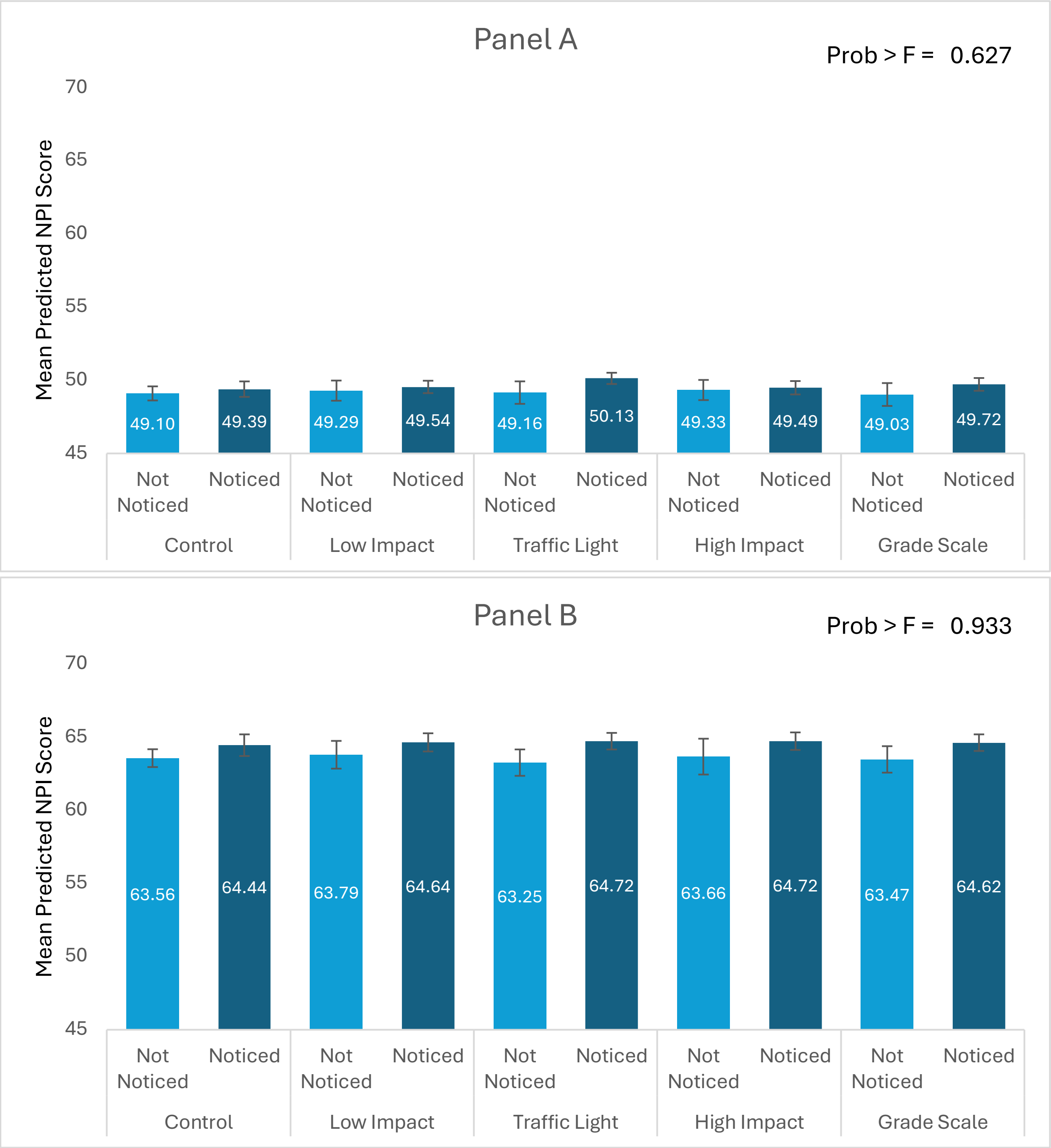

Note: Statistical significance at <0.05 with Bonferroni-Holm correction for multiple comparisons within family of outcomes.

a. Post estimation margins after weighted simple linear regression with interaction term for noticeability (yes/no)

© Wolfson JA 2026 et al. JAMA Health Forum.

Table e5. Mean Predicted Probabilities of Noticing Label, Identifying What Label was About, and Label Use by Treatment Condition [a]

	Overall	Control	Low-Impact		Traffic-Light		High-Impact		Grade-Scale	
Reported noticing label	(n=6209)	(n=1235 [19.8%])	(n=1249 [20.4%])		(n=1235 [19.0%]		(n=1243 [20.2%])		(n=1247 [20.6%])	
	4152 (66.9)	42.9 (39.2, 46.5)	**69.8 (66.4, 73.2)**	**<0.001**	**78.3 (75.3, 81.3)**	**<0.001**	**75.1 (71.9, 78.3)**	**<0.001**	**68.5 (64.9, 72.1)**	**<0.001**
Reported what the label was about [b]	(n=4152)	(n= 512 [12.7%])	(n=874 [21.3%])		(n=965 [22.3%]		(n=924 [22.6%])		(n=877 [21.1%])	
Sugar	47 (1.0)	2.6 (0.5, 4.8)	**0.8 (0.2, 1.5)**	0.04	0.8 (0.1, 1.5)	0.06	**0.7 (0.0, 1.4)**	0.04	0.9 (0.3, 1.6)	0.05
Sodium	42 (1.3)	1.9 (0.6, 3.1)	1.8 (0.6, 3.0)	0.93	1.0 (0.1, 1.8)	0.25	0.8 (-0.1, 1.7)	0.19	1.5 (0.1, 2.9)	0.70
QR code	353 (8.8)	62.0 (56.4, 67.6)	**0.3 (-0.1, 0.7)**	**<0.001**	**1.2 (0.0, 2.4)**	**<0.001**	**0.6 (0.0, 1.1)**	**<0.001**	**2.1 (0.7, 3.5)**	**<0.001**
Climate impact	3096 (73.7)	1.7 (-0.7, 4.2)	**79.2 (75.6, 82.8)**	**<0.001**	**88.7 (86.0, 91.4)**	**<0.001**	**88.1 (85.5, 90.7)**	**<0.001**	**80.3 (76.9, 83.6)**	**<0.001**
Organic	23 (0.6)	0.5 (-0.1, 1.2)	1.1 (0.0, 2.2)	0.39	0.4 (-0.2, 0.9)	0.69	0.2 (-0.1, 0.6)	0.37	0.6 (0.0, 1.1)	0.98
Healthy	126 (2.9)	2.1 (0.6, 3.6)	**4.9 (2.9, 6.9)**	0.05	2.9 (1.5, 4.2)	0.50	1.4 (0.6, 2.2)	0.37	3.0 (1.7, 4.3)	0.42
Unhealthy	57 (1.7)	4.6 (1.8, 7.4)	**0.6 (0.1, 1.0)**	**<0.001**	**1.1 (0.2, 2.1)**	0.007	**1.4 (0.4, 2.5)**	0.02	1.7 (0.6, 2.8)	0.03
None of these	220 (5.3)	11.8 (8.5, 15.2)	**6.1 (4.0, 8.2)**	0.004	**1.7 (0.7, 2.7)**	**<0.001**	**3.6 (2.1, 5.2)**	**<0.001**	**3.3 (1.8, 4.8)**	**<0.001**
I don't know/ I can't remember	188 (4.7)	12.6 (8.7, 16.6)	**5.2 (3.2, 7.2)**	**0.001**	**2.2 (1.2, 3.3)**	**<0.001**	**3.1 (1.8, 4.4)**	**<0.001**	6.6 (4.6, 8.5)	0.003
Reported using the label when deciding what to order [c]	(n=3424)	(n= 335 [9.9%])	(n=705 [20.7%])		(n=866 [24.2%]		(n=813 [24.4%])		(n=705 [20.7%])	
	1052 (29.6)	21.5 (15.4, 27.6)	24.3 (20.1, 28.5)	0.46	**38.9 (34.5, 43.2)**	**<0.001**	**30.1 (25.9, 34.3)**	0.03	27.5 (23.4, 31.7)	0.12

Note: Statistical significance at <0.05 with Bonferroni-Holm correction for multiple comparisons within family of outcomes. Bolded scores remained significant after Bonferroni-Holm correction for multiple comparisons.

a. Mean predicted probabilities from post estimation margins after weighted simple logistic regression. 95% CIs are shown in parentheses.
b. Only participants who reported noticing a label were asked to report what the label was about. The overall column show the n (%) of people reporting each item.

© Wolfson JA 2026 et al. JAMA Health Forum.

c. Only participants who reported noticing a label and correctly identified what the label told them about (control group and reported that label told them about a QR code or intervention group and reported that label told them about climate change) were asked whether they used the label when deciding what to order.

© Wolfson JA 2026 et al. JAMA Health Forum.

Table e6. Mean Predicted Probabilities of Correctly Identifying Higher Climate Impact Menu Items and Ranking Items by Climate Impact by Treatment Condition (n=6242) [a]

	**Overall** (n=6242)	**Control** (n=1252)	**Low-Impact** (n=1251)		**Traffic-Light** (n=1241)		**High-Impact** (n=1250)		**Grade-Scale** (n=1248)	
	n (%)	% (95% CI)	% (95% CI)	*p*	% (95% CI)	*p*	% (95% CI)	*p*	% (95% CI)	*p*
Correctly Identified Menu Items with Higher Climate Impact [b]	3,436 (54.3)	**31.01 (27.67, 34.35)**	**50.23 (46.56, 53.89)**	**<0.001**	**66.07 (62.64, 69.50)**	**<0.001**	**64.10 (60.61, 67.59)**	**<0.001**	**60.55 (56.88, 64.22)**	**<0.001**
Correctly Ranked Tofu, Chicken, and Beef from Lowest to Highest Climate Impact [c]	4181 (65.2)	**57.45 (53.85, 61.05)**	**66.27 (62.80, 69.74)**	**0.001**	**70.52 (67.11, 73.92)**	**<0.001**	62.54 (58.91, 66.16)	0.05	**69.24 (65.72, 72.76)**	**<0.001**

Note: Statistical significance at <0.05 with Bonferroni-Holm correction for multiple comparisons within family of outcomes. Bolded scores remained significant after Bonferroni-Holm correction for multiple comparisons.

a. Mean predicted probabilities from post estimation margins after weighted simple logistic regression
b. Probability of correctly identifying that chicken sandwich has lower climate impact compared to whopper at Burger King AND correctly identifying that black forest ham sandwich has lower climate impact than roast beef sandwich at Subway
c. Probability of correctly ranking climate impact of tofu, chicken, and beef from 1-3, where 1 is lowest climate impact and 3 is highest climate impact.

© Wolfson JA 2026 et al. JAMA Health Forum.

Figure e2. Mean Predicted Nutrition Profile Index Score of Selected Main Item at the Burger Restaurant (Panel A) and the Sandwich Restaurant (Panel B) by Treatment Condition (n=6210) [a]

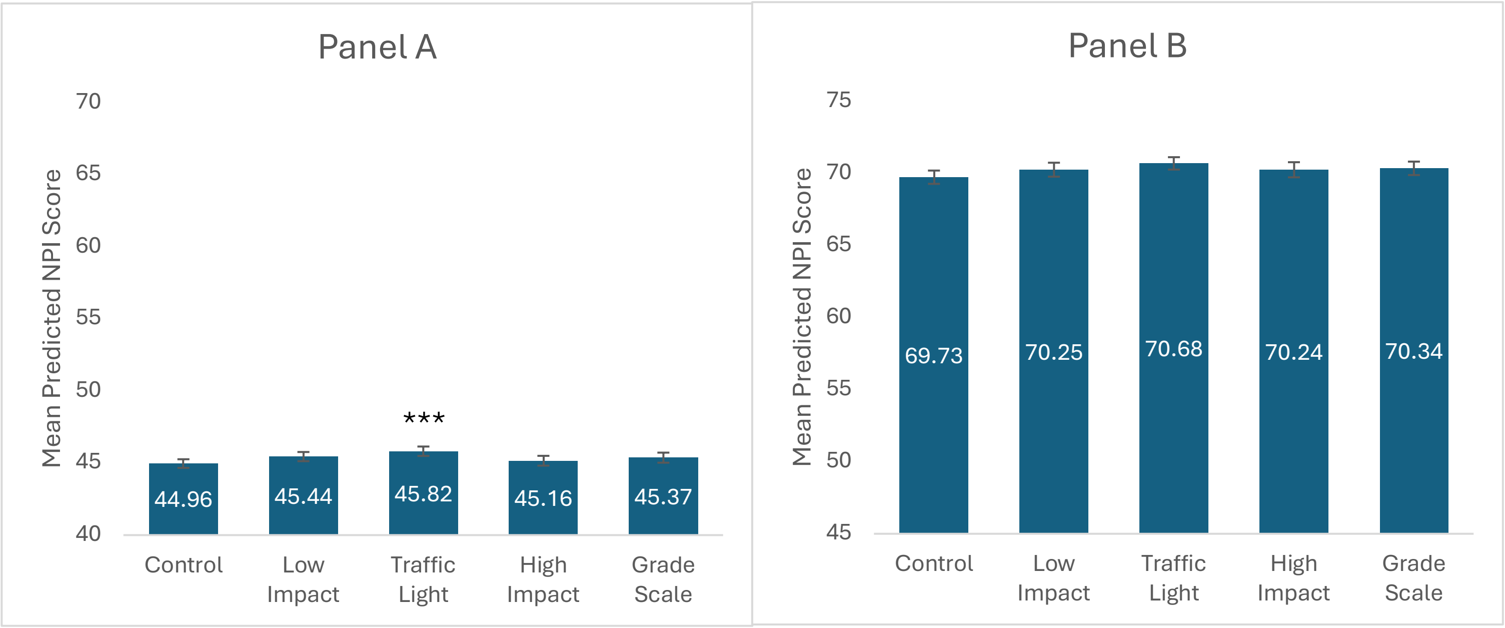

Note: Statistical significance at <0.05 with Bonferroni-Holm correction for multiple comparisons within family of outcomes. *** Indicates p<0.001.

a. Post estimation margins after weighted simple linear regression

© Wolfson JA 2026 et al. JAMA Health Forum.

Figure e3. Predicted Probability of Selecting Sustainable Item at the Burger Restaurant (Panel A) and the Sandwich Restaurant (Panel B) by Treatment Condition (n=6210) [a]

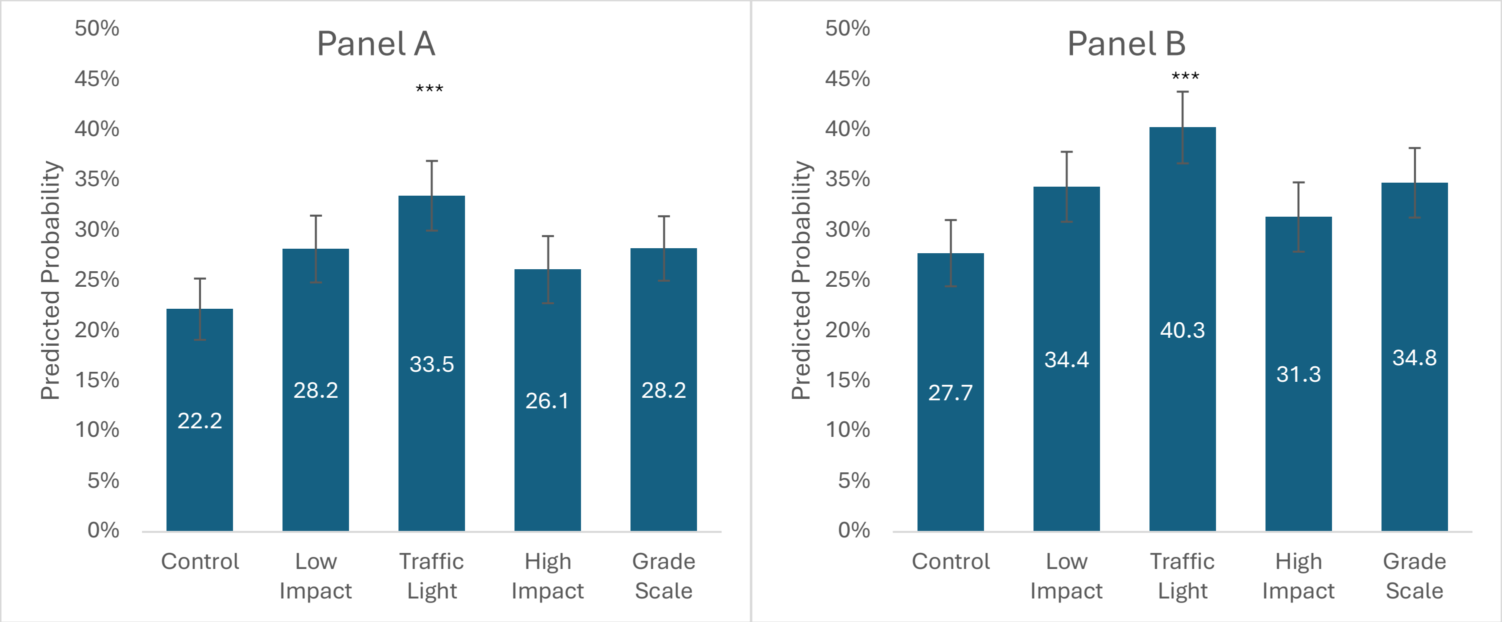

Note: Statistical significance at <0.05 with Bonferroni-Holm correction for multiple comparisons within family of outcomes. *** Indicates p<0.001.

a. Post estimation margins after weighted simple logistic regression

© Wolfson JA 2026 et al. JAMA Health Forum.

Figure e4. Predicted Probability of Selecting Item Containing Red Meat the Burger Restaurant (Panel A) and the Sandwich Restaurant (Panel B) by Treatment Condition (n=6210) [a]

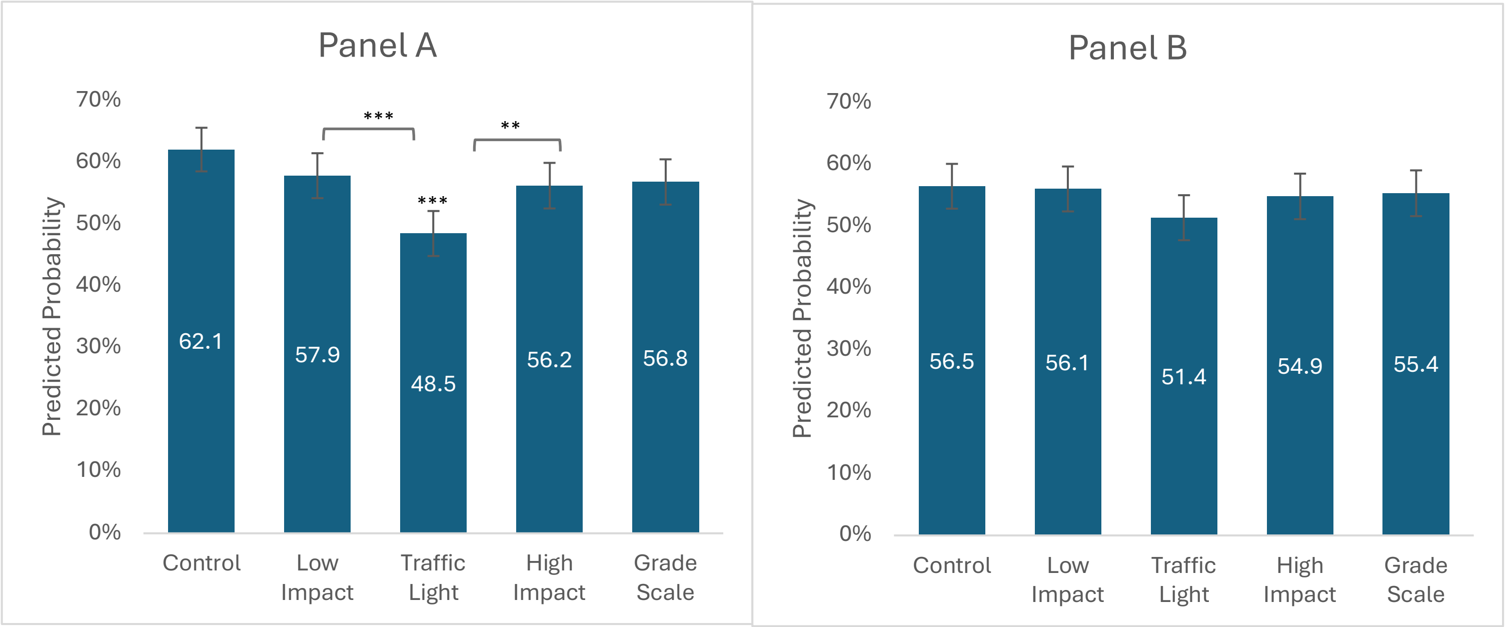

Note: Statistical significance at <0.05 with Bonferroni-Holm correction for multiple comparisons within family of outcomes. ** Indicates p<0.01. *** Indicates p<0.001.

a. Post estimation margins after weighted simple logistic regression

© Wolfson JA 2026 et al. JAMA Health Forum.

Figure e5. Predicted Probability of Selecting a Sugar Sweetened Beverage at the Burger Restaurant (Panel A) and the Sandwich Restaurant (Panel B) by Treatment Condition (n=6210) [a]

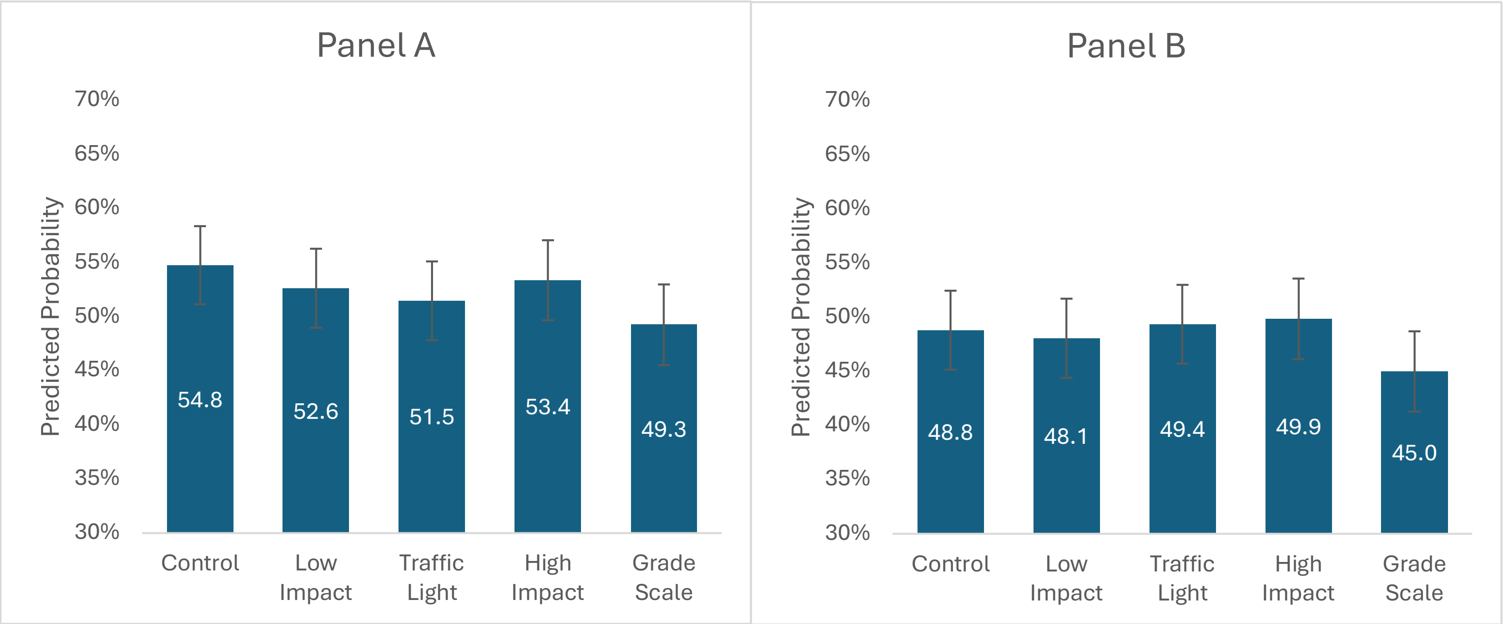

Note: Statistical significance at <0.05 with Bonferroni-Holm correction for multiple comparisons within family of outcomes.

a. Post estimation margins after weighted simple logistic regression

© Wolfson JA 2026 et al. JAMA Health Forum.

Figure e6. Mean Predicted Cost of selected Meals at the Burger Restaurant (Panel A) and the Sandwich Restaurant (Panel B) by Treatment Condition (n=6210) [a]

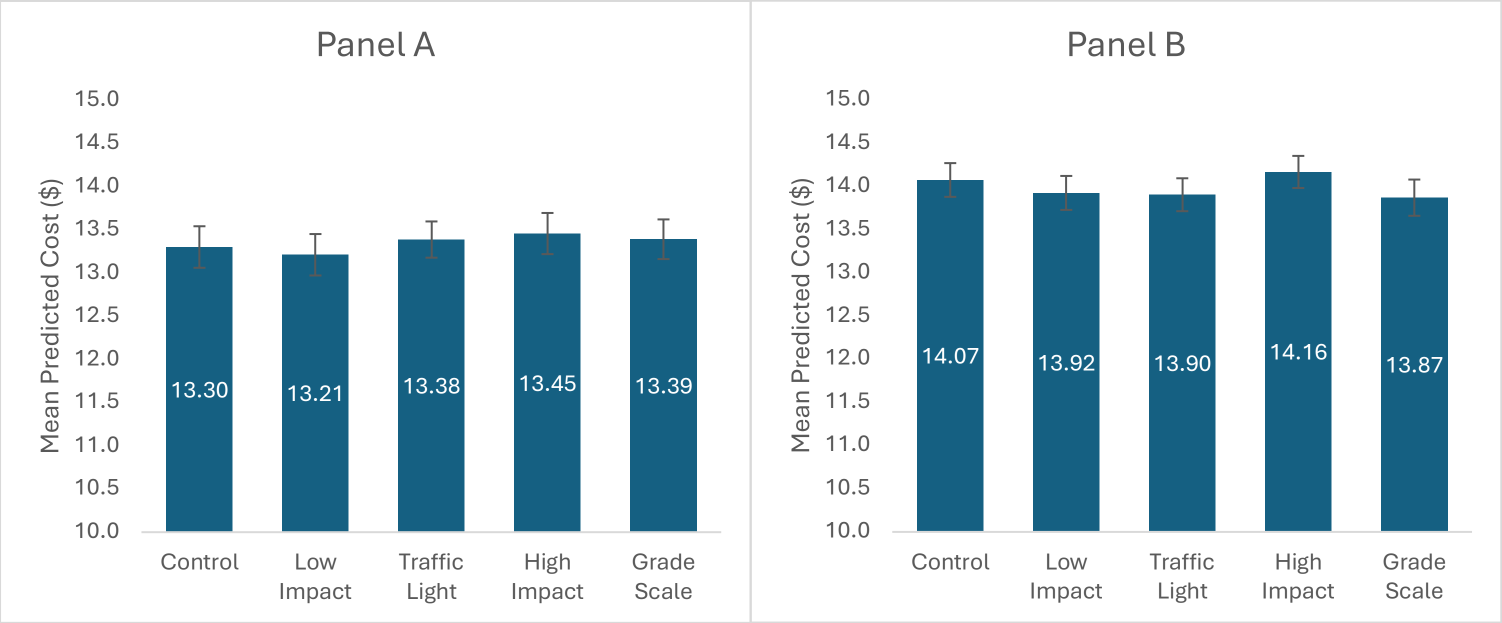

Note: Statistical significance at <0.05 with Bonferroni-Holm correction for multiple comparisons within family of outcomes.

a. Post estimation margins after weighted simple linear regression

© Wolfson JA 2026 et al. JAMA Health Forum.

Table e7. Summary Table of Key Results Across Label Conditions.

Label	Overall		Burger Restaurant		Sandwich Restaurant	
	% Noticed	Mean PME	NPI	GHGE	NPI	GHGE
SCAN HERE	42.9	1.88	49.25	21.1	63.94	19.4
LOW CLIMATE IMPACT	69.8***	2.22***	49.47	20.8**	64.38	18.3**
HIGH CLIMATE IMPACT<br>MED CLIMATE IMPACT<br>LOW CLIMATE IMPACT	78.3***	2.62***	49.93***	17.9***	64.40	13.6***
HIGH CLIMATE IMPACT	75.1***	2.60***	49.45**	21.4**	64.45	17.6**
C A B C D F CLIMATE GRADE	68.5***	2.56***	49.50**	20.1	64.26	15.9**

Note: NPI= Nutrition Profile Index; GHGE= Greenhouse Gas Emissions; PME= Perceived Message Effectiveness. Statistical significance at <0.05 with Bonferroni-Holm correction for multiple comparisons of four label conditions. Meals exclude beverages.
** Indicates different from the Traffic Light Label at p<0.01.
*** Indicates different from control label at p<0.001.

© Wolfson JA 2026 et al. JAMA Health Forum.

4. Menu Images for different label conditions

Note. Menus have been modified to fit on these pages.

Main Item Menus with Control (QR Code) Labels

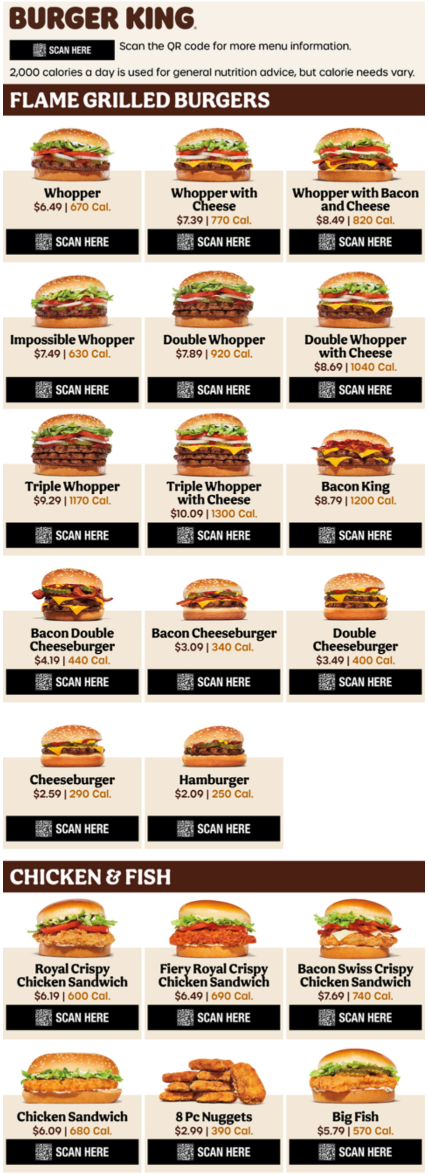

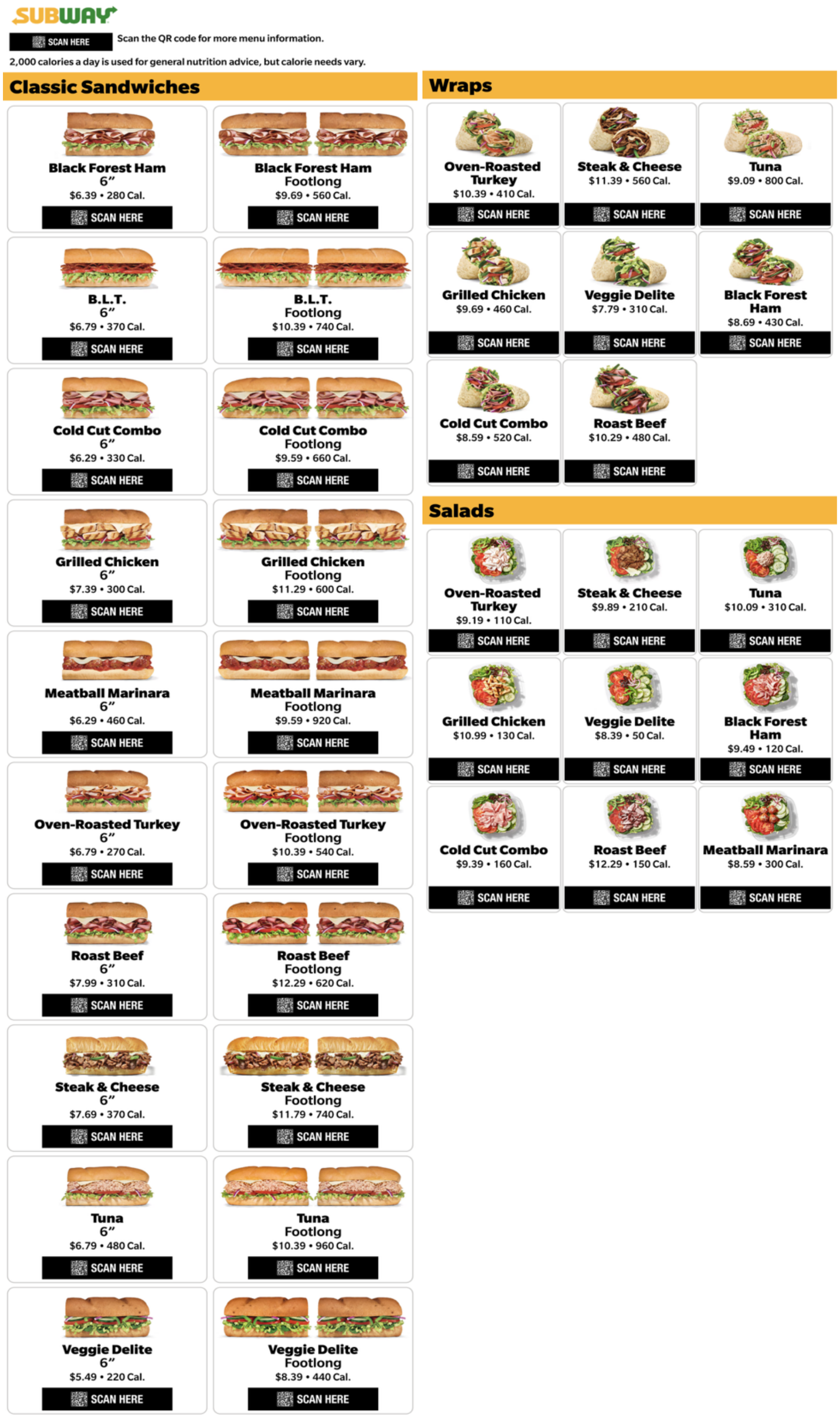

Main Item Menus with Low-Impact Labels

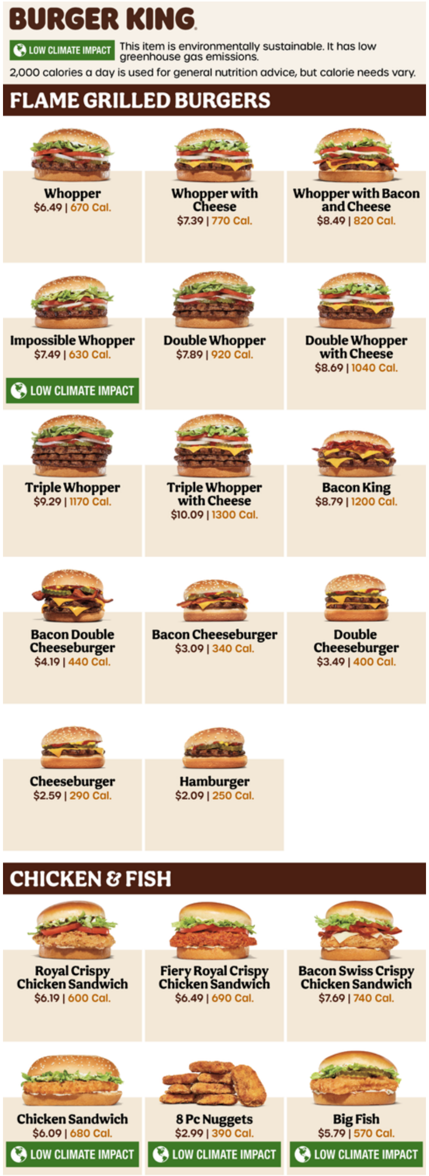

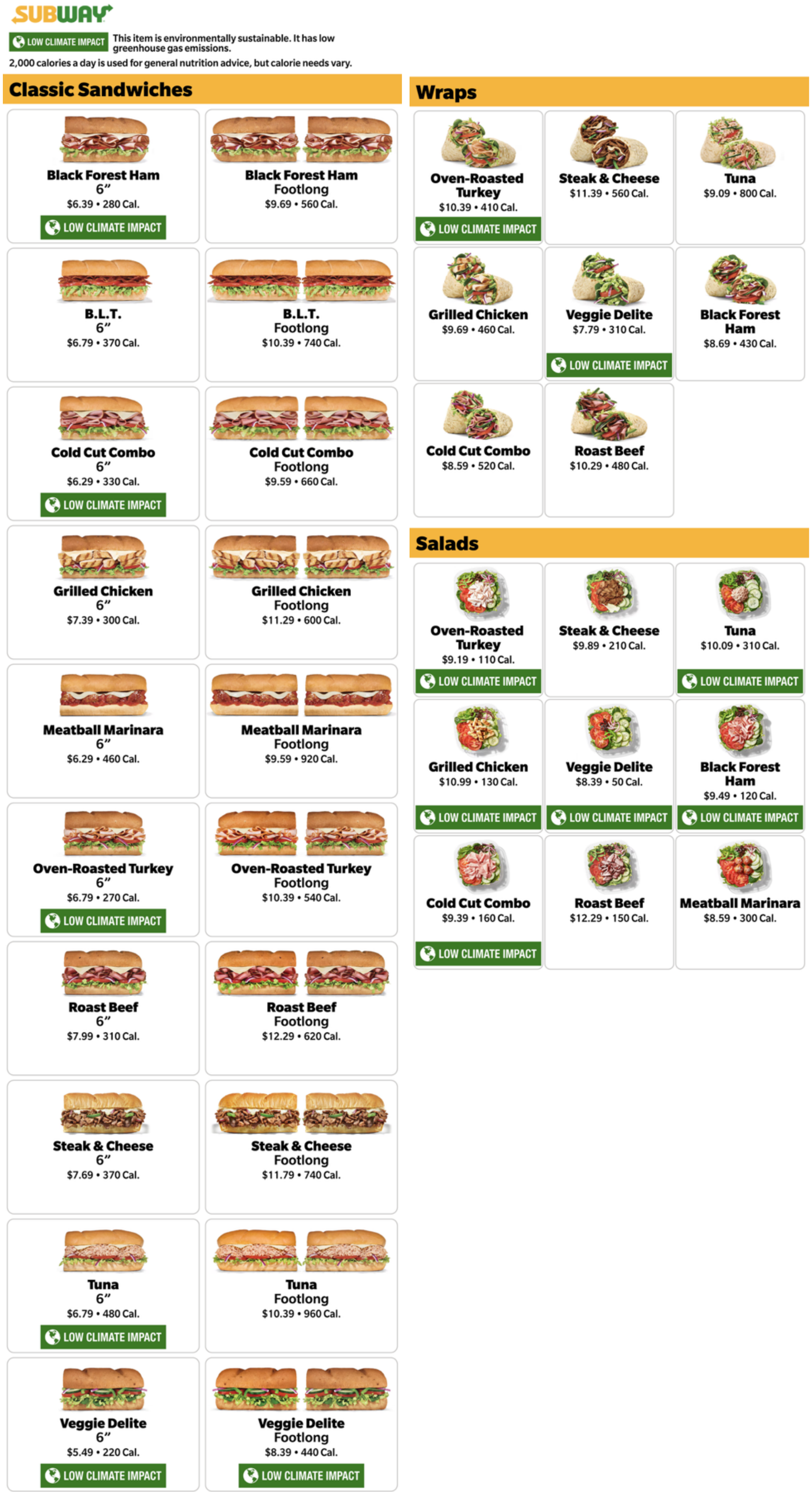

© Wolfson JA 2026 et al. JAMA Health Forum.

Main Item Menus with High-Impact Labels

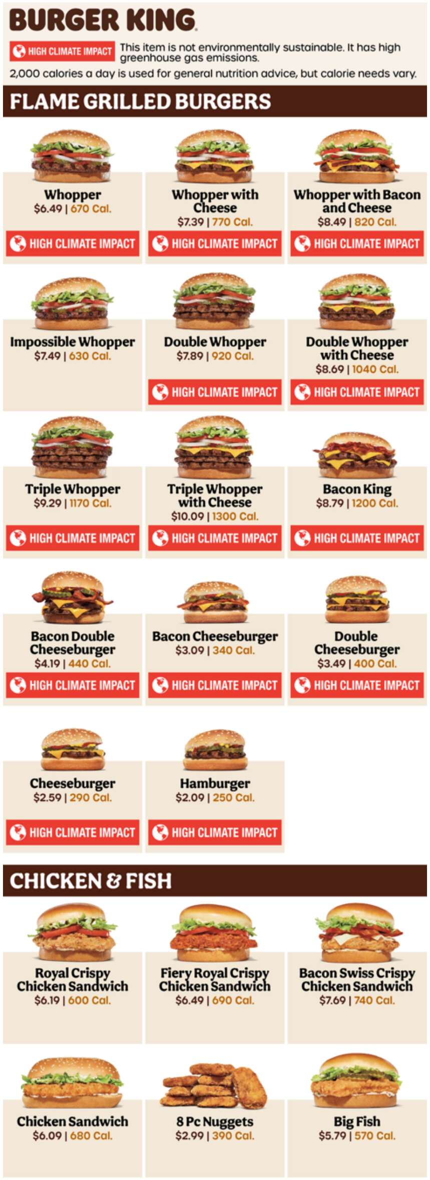

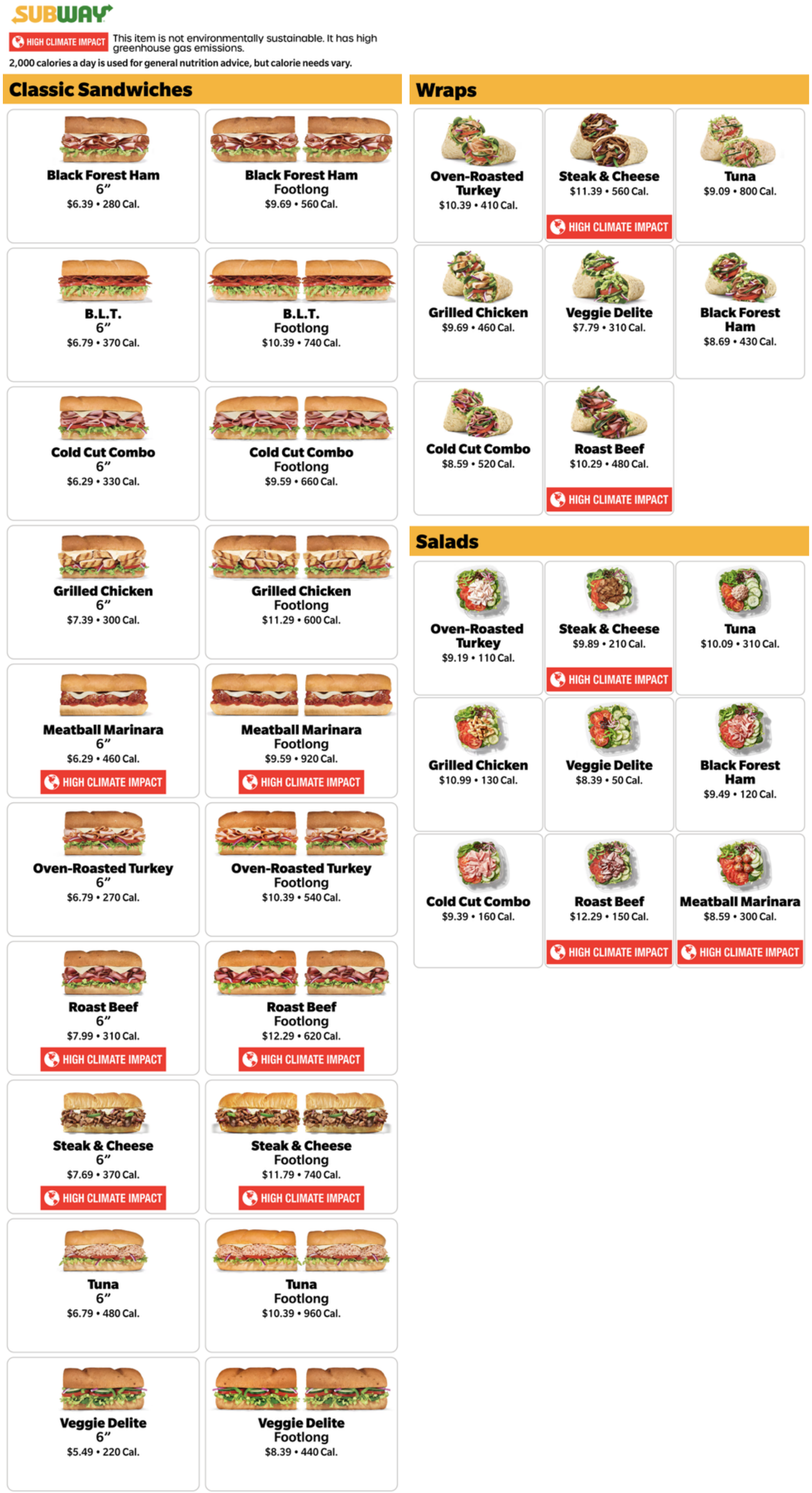

© Wolfson JA 2026 et al. JAMA Health Forum.

Main Item Menus with Traffic-Light Labels

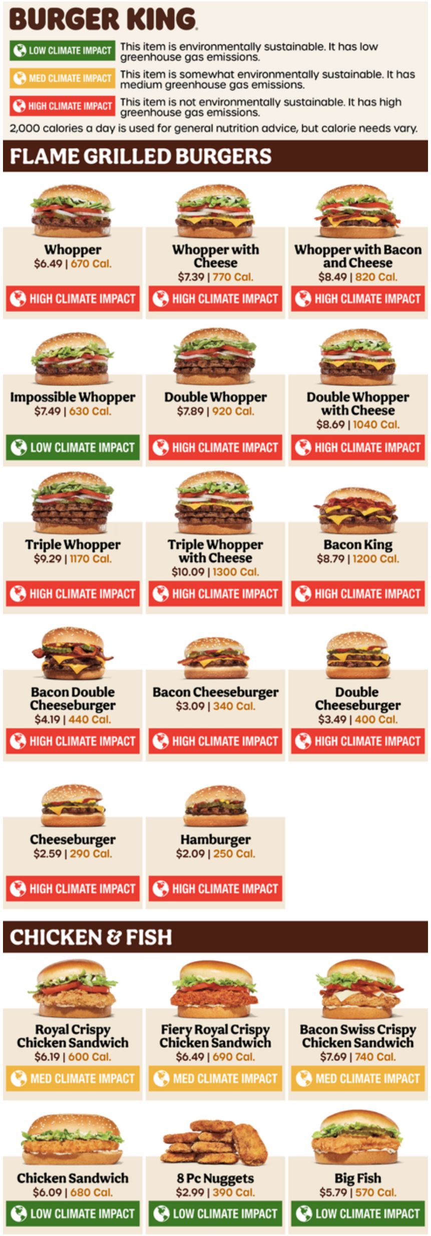

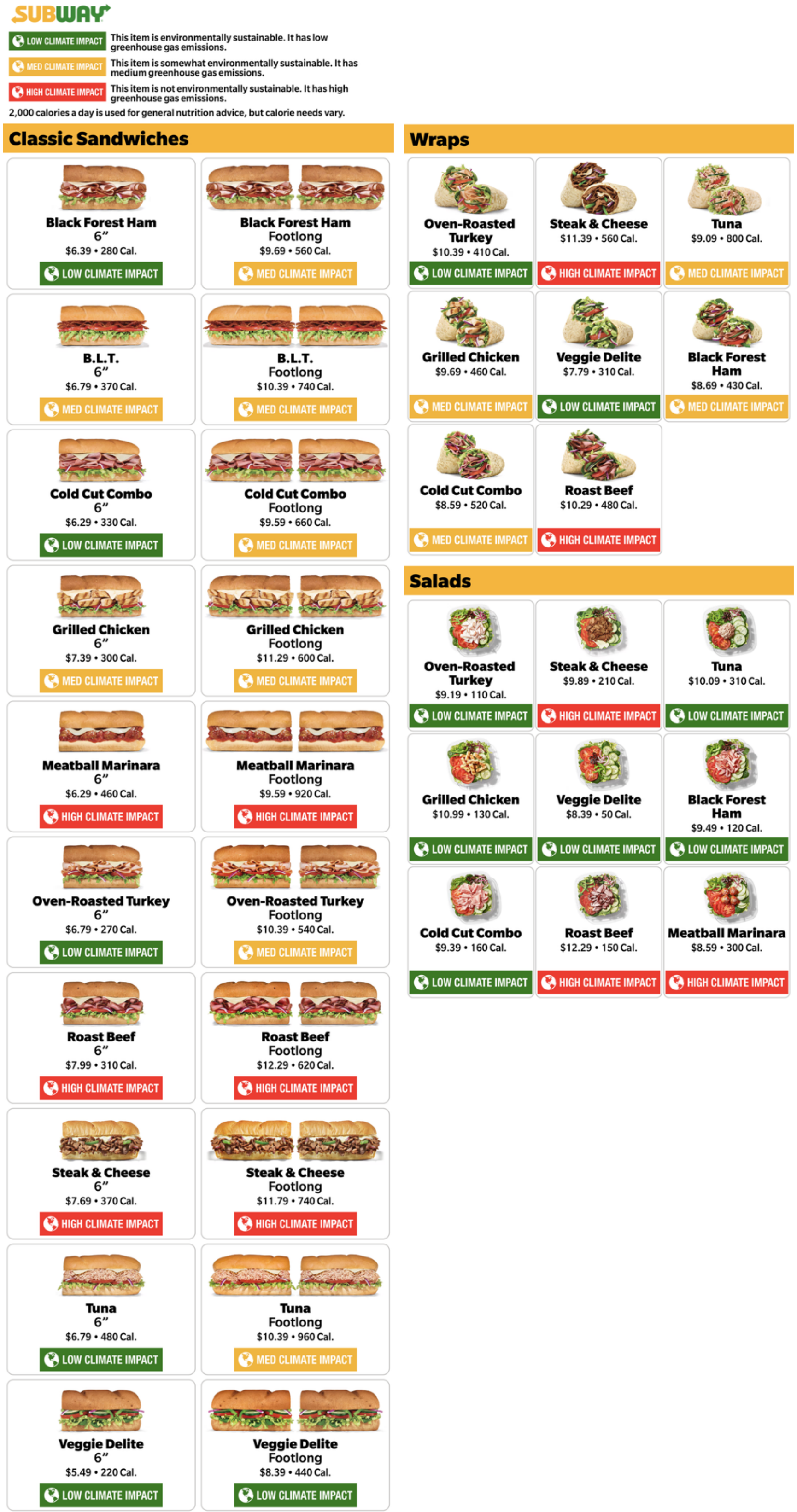

© Wolfson JA 2026 et al. JAMA Health Forum.

Main Item Menus with Grade-Scale Labels

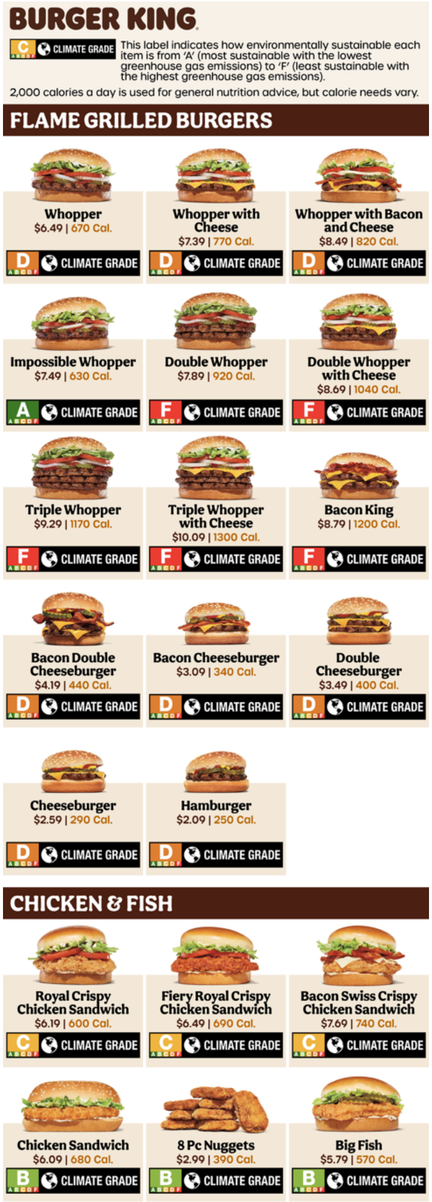

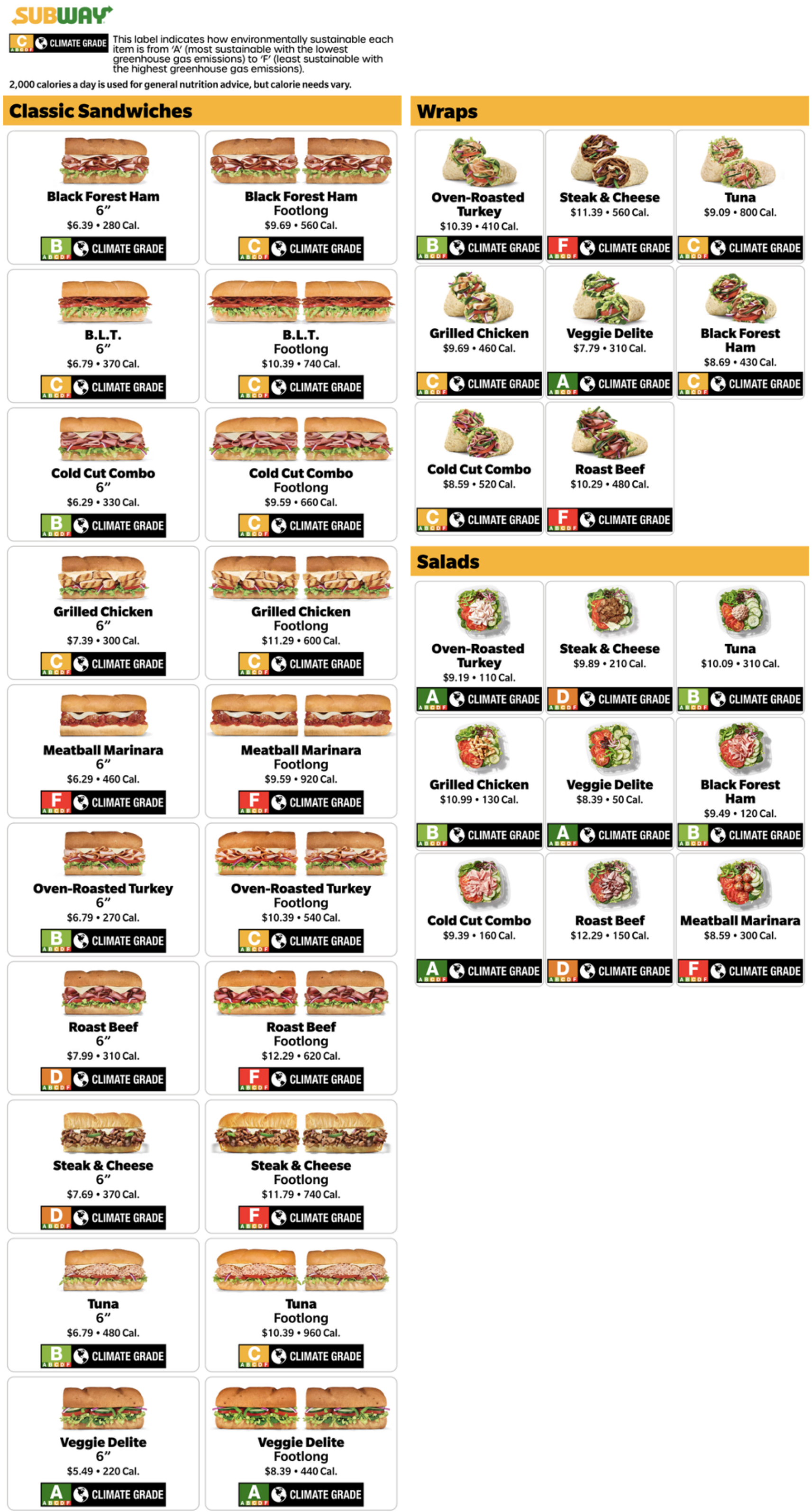

© Wolfson JA 2026 et al. JAMA Health Forum.

Side, Dessert, and Drink Menus

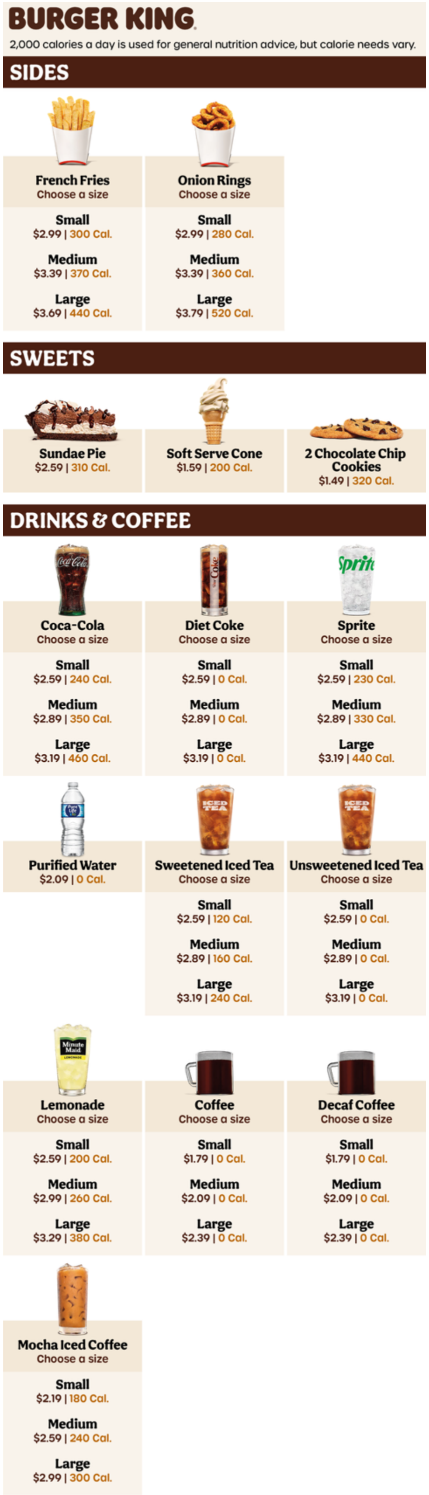

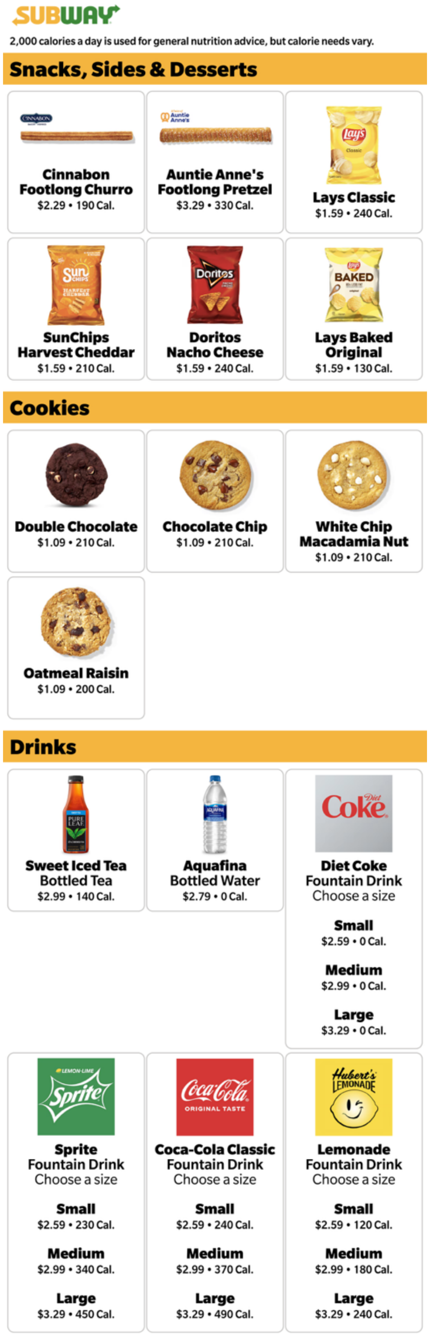

© Wolfson JA 2026 et al. JAMA Health Forum.
