# Supplementary material for: Eco-Focused Menu Labels on Full Meal Orders From Fast-Food Restaurants: A Randomized Clinical Trial
Source: JAMA Health Forum. 2026 Jul 10;7(7):e262108. doi: 10.1001/jamahealthforum.2026.2108 (PMC13355149; doi:10.1001/jamahealthforum.2026.2108)
Supplement: Supplement 3. — Data Sharing Statement [file jamahealthforum-e262108-s003.pdf]

Data Sharing Statement

Wolfson. Eco-Focused Menu Labels on Full Meal Orders From Fast-Food Restaurants. *JAMA Health Forum*. Published July 10, 2026. doi:10.1001/jamahealthforum.2026.2108

Data
Additional Information: Clinicaltrials.gov Identifier: NCT06909019
Data available: Yes
Data types: Deidentified participant data, Data dictionary
How to access data: Contact the corresponding author at jwolfso7@jhu.edu. At a later date data will be shared on the Open Science Framework website
When available: With publication

Supporting Documents
Document types: None

Additional Information
Who can access the data: Anyone with a request approved by the study team.
Types of analyses: Any approved purpose.
Mechanisms of data availability: After approval.
Any additional restrictions: None
